# *Terpene Synthase-b* and *Terpene Synthase-e/f* Genes Produce Monoterpenes for *Phalaenopsis bellina* Floral Scent

**DOI:** 10.3389/fpls.2021.700958

**Published:** 2021-07-14

**Authors:** Hsin Huang, Yi-Wei Kuo, Yu-Chen Chuang, Ya-Ping Yang, Li-Min Huang, Mei-Fen Jeng, Wen-Huei Chen, Hong-Hwa Chen

**Affiliations:** ^1^Department of Life Sciences, National Cheng Kung University, Tainan, Taiwan; ^2^Orchid Research and Development Center, National Cheng Kung University, Tainan, Taiwan; ^3^Institute of Tropical Plant and Microbial Sciences, National Cheng Kung University, Tainan, Taiwan

**Keywords:** *Phalaenopsis*, monoterpene, floral scent, terpene synthase, geraniol, linalool, orchid, recombinant protein

## Abstract

Orchids are the most species-rich plants and highly interactive with pollinators via visual or olfactory cues. Biosynthesis and emission of volatile organic compounds (VOCs) to the atmosphere facilitate the olfactory cues and ensure successful pollination. *Phalaenopsis bellina* is a scented orchid with monoterpenes as major VOCs, comprising linalool, geraniol, and their derivatives. Comparative transcriptomics analysis identified four *terpene synthase-b* (*TPS-b*) genes and two *TPS-e/f* genes with differential gene expression between scented and scentless *Phalaenopsis* species. Here, we confirmed their differential expression between scented and scentless *Phalaenopsis* orchids and excluded one *TPS-b* candidate. We analyzed the temporal and spatial expression and functionally characterized these *TPSs*. Both *TPS-b* and *TPS-e/f* genes showed an increased expression on blooming day or 3 days post-anthesis (D + 3) before the optimal emission of floral scent on D + 5, with especially high expression of *PbTPS5* and *PbTPS10*. The *TPS-b* genes are expressed exclusively in reproductive organs, whereas the *TPS-e/f* genes are expressed in both reproductive and vegetative organs. *In planta* functional characterization of both *PbTPS5* and *PbTPS10* in tobacco and scentless *Phalaenopsis* plants did not produce terpenoids. Further ectopic expression in scented *Phalaenopsis* cultivar *P.* I-Hsin Venus showed that linalool was the main product, with *PbTPS10* displaying 3-fold higher activity than *PbTPS5.* On *in vitro* enzyme assay with purified recombinant TPS-b proteins ectopically expressed in *Escherichia coli*, geraniol was the product catalyzed by *PbTPS5* and *PbTPS9*. *PbTPS3* was a linalool/(β)-cis-ocimene synthase and *PbTPS4* a linalool synthase. In conclusion, both *TPS-b* and *TPS-e/f* enzymes orchestrated floral monoterpene biosynthesis in *P. bellina*.

## Introduction

Orchidaceae is one of the most species-rich families in angiosperms and highly interactive with their pollinators. Interaction between pollinators and orchids has played a major role in orchid evolution (Schiestl et al., 2000; Sanguinetti et al., 2012). In Orchidaceae, both visual and olfactory captures are crucial for successful pollination. For olfactory capture, orchids emit floral volatile organic compounds (VOCs), including terpenoids, phenylpropanoids, and benzoids, into the atmosphere. Floral VOCs have been characterized in several orchids, including monoterpenes of linalool and geraniol in *Phalaenopsis bellina* (Hsiao et al., 2006). Emission of the floral scent is developmentally regulated: It is undetectable on blooming day or with flower buds, starts to be detected on 3 days post-anthesis (D + 3), and is optimized on D + 5. It decreases as the flower becomes senescent (Hsiao et al., 2006).

Terpenes represent the largest group of plant secondary metabolites. Terpenoids have many diverse structures, with various carbon skeletons and a large assortment of functional groups. The biosynthesis of a terpene starts from the formation of the basic C5 units, isopentenyl diphosphate (IPP), and its allylic isomer, dimethylallyl diphosphate (DMADP). IPP is synthesized via two independent pathways: the mevalonate (MVA) pathway in the cytosol and methylerythritol phosphate (MEP) pathway in the plastids. The action of various short-chain prenyltransferases produces direct precursors for terpene synthases (TPSs): monoterpene synthase (MTPS) uses geranyl diphosphate (GDP) as a substrate for producing monoterpenes (C10), sesquiterpene synthase (STPS) uses farnesyl diphosphate (FDP) as a substrate for the biosynthesis of sesquiterpenes (C15), and diterpene synthase (DTPS) uses geranylgeranyl diphosphate (GGDP) as a substrate for producing diterpenes (C20) (Spitzer-Rimon et al., 2010; Colquhoun et al., 2011). Both MTPS and DTPS in the MEP pathway are present in the plastids for monoterpene and diterpene biosynthesis, whereas STPS in the MVA pathway is present in the cytoplasm for sesquiterpene biosynthesis (Lichtenthaler et al., 1997).

*TPS* genes are a large family classified into seven major subfamilies, namely, *TPS-a* to *TPS-h* (Chen et al., 2011). The *TPS-c* subfamily is the original subfamily and contains copalyl diphosphate synthases (CPSs), CPS/kaurene synthases (CPS/KSs), and DTPSs. *TPS-a* and *TPS-b* subfamilies are the angiosperm-specific subfamilies, and they contain STPSs and MTPSs, respectively (Bohlmann et al., 1998; Chen et al., 2011). The *TPS-d* subfamily is for gymnosperms MTPS and DTPS. The *TPS-g* subfamily lacks the RRX_8_W motif for producing acyclic monoterpene and sesquiterpene. The *TPS-h* subfamily is identified as DTPS in *Selaginella moellendorffii* (Chen et al., 2011). The *TPS-e/f* subfamily contains KSs, MTPSs, STPSs, and DTPSs. Diverse functions have been reported for *TPS-e/f* enzymes, including linalool synthase in *Clarkia breweri* (Dudareva et al., 1996), geranyllinalool synthase in *Arabidopsis thaliana*, and farnesene synthase in kiwifruit (Nieuwenhuizen et al., 2009). The *TPS-f* subfamily is probably dicot-specific, whereas the *TPS-e* subfamily is not (Chen et al., 2011).

*Phalaenopsis bellina* is a native *Phalaenopsis* species in Borneo, Sarawak, and a common breeding parent for scented cultivars with a monoterpene phenotype. It has linalool, geraniol, and their derivatives as the major floral VOCs (Hsiao et al., 2006). The combination of bioinformatics and genomics identified PbGDPS as the key enzyme in orchid floral scent biosynthesis based on its high enrichment in the scented *P. bellina* but not scentless *Phalaenopsis equestris* and *Phalaenopsis aphrodite* subsp. *formosana* (hereafter *Phalaenopsis aphrodite*) (Hsiao et al., 2008; Chuang et al., 2018a). PbGDPS exhibits a dual prenyltransferase activity, producing GDP and FDP as the products, but lacks the DD(X)_2__–__4_D domain (Hsiao et al., 2008). The flower-specific expression profile of *PbGDPS* is concomitant with the emission pattern of monoterpenes on D + 5 (Hsiao et al., 2006). *PbGDPS* is the first enzyme with substantially differential expression between *P. bellina* and *Phalaenopsis aphrodite*, followed by several *TPS* genes in the monoterpene biosynthesis pathway (Chuang et al., 2018b). Comparative transcriptomics revealed the upregulated expression of *TPS-e/f* as well as *TPS-b* genes in *P. bellina* as compared with the scentless *Phalaenopsis aphrodite* (Supplementary Figure 1B).

Here, we confirm the differential gene expression of these *TPS* genes between *P. bellina* and *Phalaenopsis aphrodite*, illustrate the temporal and spatial gene expression, and provide a functional characterization of the TPSs in *TPS-b* and *TPS-e/f* subfamilies to further understand floral monoterpene biosynthesis in *Phalaenopsis* orchids. Functional characterization was revealed by both *in planta* and *in vitro* enzyme activity assays with transient overexpression and purified recombinant proteins ectopically overexpressed in *Escherichia coli*, respectively. These results suggest that both *TPS-b* and *TPS-e/f* enzymes contribute to monoterpene biosynthesis in the *P. bellina* floral scent.

## Materials and Methods

### Plant Materials

*Phalaenopsis bellina* was from the Ming-Hui Orchids Nursery (Changhua, Taiwan) and Quan-Ya Orchid Nursery (Tainan, Taiwan) (Supplementary Figure 2A), *P.* I-Hsin Venus (hereafter Venus) was from I-Hsin Biotechnology Corp. (Chiayi, Taiwan) (Supplementary Figure 2B), and *Phalaenopsis aphrodite* and *P.* Sogo Yukidian “V3” (hereafter V3) were from Taiwan Sugar Corp. (Tainan, Taiwan). All *Phalaenopsis* plants were grown in the greenhouse at the National Cheng Kung University under natural light and controlled temperature from 27 to 30°C with 80% humidity. Venus, a commercial scented hybrid (Supplementary Figure 2B), was selected because it has mild levels of floral linalool emission. The genetic background of Venus is as follows: *Phalaenopsis amboinensis* (25%), *Phalaenopsis equestris* (25%), *Phalaenopsis venosa* (18.7%), *Phalaenopsis violacea* (15.6%), *Phalaenopsis amabilis* (10.4%), *Phalaenopsis lueddemanniana* (3.1%), and *Phalaenopsis aphrodite* (2.1%) via ORCHIDEYA.CA^1^.

*Nicotiana tabacum* seeds were obtained from Dr. Ching-Chun Chang’s laboratory (Department of Biotechnology and Bioindustry Sciences, NCKU) and germinated directly in soil and grown under a 16-h day/8-h night photoperiod with temperatures set at 27°C. Humidity was adjusted to 70%.

### Phylogenetic Analysis

For TPS phylogenetic analysis, previously studied TPS sequences were downloaded from NCBI isolated from other plant species, and the full-length sequences were analyzed with the putative TPSs isolated from *P. bellina* and *Phalaenopsis aphrodite* transcriptomes. Information on TPSs used for phylogenetic analysis is in Supplementary Tables 1, 2. The alignment was performed by using ClustalW of the Molecular Evolutionary Genetics Analysis (MEGA7) program. The phylogenetic tree was constructed by using the neighbor-joining method with the Jones–Taylor–Thornton model and pairwise deletion with 1,000 bootstrap replicates.

### Quantitative Real-Time PCR and Gene Expression Clustering

Total RNA was extracted from seven stages of *P. bellina* flowers, starting from D − 1 to D + 17, as described in the study by Chuang et al. (2018b). After DNA contamination was removed by DNase I (New England Biolabs, Beverly, MA, United States), reverse transcription of DNA to cDNA involved using SuperScript III (Invitrogen, Carlsbad, CA, United States). Primers were designed by using Primer Express 3.0 (Applied Biosystems, Foster City, CA, United States). Quantitative Reverse transcription-PCR (RT-PCR) (qRT-PCR) involved using a StepOne Real Time PCR System and SYBR Green kit (Applied Biosystems, Foster City, CA, United States) as described in the study by Chuang et al. (2018b). Several reference genes widely used in *Phalaenopsis* orchids were identified by a local BLASTN search (Chen et al., 2005; Hsiao et al., 2008; Hsieh et al., 2013; Hsu et al., 2015). *Actin1* had equivalent levels in the RNA-seq analysis of the *P. bellina* transcriptome and microarray data for *Phalaenopsis aphrodite* (Chuang et al., 2018b), so *PbActin1* in *P. bellina* was selected as an internal calibrator for further analysis. The expression of all genes was normalized by the reference gene *PbActin1*. Calculation of mean and error bars was based on three replicates. The temporal gene expression experiments for both *TPS-e/f* and *TPS-b* were performed in different years with *P. bellina* from different orchid nurseries. *P. bellina* from various orchid nurseries have different scent emission profiles: Some last longer, and others are shorter but with the same volatile compounds. The floral scent emission for *P. bellina* lasted longer for *TPS-b* gene expression analysis, so we analyzed *TPS-b* gene expression from bud to D + 17, whereas those used for *TPS-e/f* gene expression analysis has a shorter scent emission profile, so it was analyzed from 5 days before anthesis (D − 5) to D + 10.

### Transient Expression in *Nicotiana tabacum*

The modified pCAMBIA1304 vector containing *PeMYB2*, which contributes to the red color formation in *Phalaenopsis* spp. flowers (Hsu et al., 2015), was used as a binary vector for the construction of transient expression in *N. tabacum* and *Phalaenopsis* orchids. *TPS* genes were constructed into the modified binary vector, and then the recombinant vectors containing inserted *TPS* genes (dubbed pCAMBIA1304-*TPS*) were transformed into *Agrobacterium tumefaciens* strain GV3101 by electroporation. The transformed *Agrobacteria* were spread on LB agar plates containing rifampicin (10 μg/ml), gentamycin (25 μg/ml), and kanamycin (50 μg/ml). The plates were incubated for 2 days at 28°C, and colonies were tested for insertion by colony PCR with ProTaq DNA Polymerase (Protech Technology Enterprise, Taiwan). A transformed single colony was used to inoculate a 5-ml culture of LB medium plus appropriate antibiotics, and cells were grown for 24 h at 28°C and 220 rpm. This pre-culture was used to inoculate a 100-ml culture and grown for about 4 h at 28°C to OD_600_ = 0.8–1.0. *Agrobacteria* were harvested by centrifugation at 5,000 × *g* and 4°C for 15 min, then resuspended in infiltration medium (Murashige and Skoog medium, 1 mM acetosyringone) to OD_600_ = 0.4, and incubated for 2–4 h at room temperature. Diluted *Agrobacteria* was injected into the abaxial side of the three youngest *N. tabacum* leaves that were > 1 cm by using a 1-ml syringe. Transformed plants were maintained in a growth chamber at 28°C under long-day conditions (16 h/8 h for light/dark), and gas chromatography–mass spectrometry (GC-MS) analysis was performed at 5–7 days post-infiltration.

### Transient Overexpression in *Phalaenopsis* Orchids

The expression plasmid, pCAMBIA1304-*TPS* as described above, was transformed into *Agrobacterium tumefaciens* strain *EHA105* by electroporation. The growth of agrobacteria was as described above. The transformed *Agrobacteria* was injected into the adaxial side of sepals and petals at different floral stages (flower bud or just bloomed flower) by using a 0.5-ml insulin syringe. Transformed plants were maintained at 25°C, and GC-MS analysis was performed at 5 dpi. Because the constructs containing *PeMYB2* gene activated the anthocyanin pathway, red color formation in transformed flowers was considered a positive transformation in *Phalaenopsis* orchids.

### Expression and Purification of Recombinant Proteins in *Escherichia coli*

For ectopic overexpression in *E. coli*, the open reading frames of PbTPSs were amplified and cloned into the expression vector pET-28b (Novagen, Madison, WI, United States), which has a His-tag on both the N- and C-termini of the inserted fragment. The construct was introduced into *E. coli* C41 (DE3) pLysS strain (Invitrogen, Carlsbad, CA, United States). Sequence analysis and protein structure modeling confirmed that no mutant occurred near the protein active site on DNA amplification. A single colony of the constructs was incubated in 5 ml LB medium with 50 μl/ml kanamycin overnight at 37°C. Then, the culture was transferred to 750 ml LB medium with 50 μl/ml kanamycin, incubated at 37°C to OD_600_ = 0.4–0.6, and induced with 0.4 mM isopropyl β-D-1-thiogalactopyranoside (IPTG) and grown under 16°C for 22 h. Cells were pelleted by centrifugation and suspended in extraction buffer (0.3 M NaCl, 50 mM HEPES, pH 7.4), then disrupted by sonication (SONICS, VC750, Newtown, CT, United States). Cell extracts were filtered through a 0.45-μm filter. Recombinant proteins were purified by using HiTrap TALON crude (GE Healthcare, Abingdon, United Kingdom) with different concentrations of imidazole solution (0.3 M NaCl, 50 mM HEPES, 5 mM/150 mM imidazole, pH 7.4). Protein expression was verified on Coomassie Blue-stained SDS–PAGE gel and Western blot analysis with 1/10,000 diluted mouse anti-histidine monoclonal antibody (Roche Diagnostics, Penzberg, Germany).

### Enzyme Activity Assay of Purified Recombinant Proteins

The affinity-purified recombinant proteins were concentrated and incubated with assay buffer (HEPES, pH7.4, 0.1 M KCl, 10 mM MgCl_2_, 10 mM DTT, 10% glycerol) by using a 30 MWCO PES Vivaspin 50-ml concentrator (Sartorius Stedium Biotech, Goettingen, Germany). For functional characterization, recombinant protein was added to a single vial and diluted with assay buffer to a total volume of 500 μl, then incubated with GDP (2.5 μM) for 1 h at 30°C.

### Volatile Collection and GC-MS Analysis

The floral volatiles of the *Phalaenopsis* orchids were collected by using a scent-extracting apparatus (Chuang et al., 2017). The scent emission pattern of *Phalaenopsis* orchids was measured from a single flower, with three biological replicates. The volatiles collected were eluted by hexane and identified by gas chromatography/high-resolution mass spectrometry (GC/HRMS) at the NCKU Instrument Center, as described in the study by Hsiao et al. (2006). As a negative control, metabolites originating from the scent-extracting apparatus were analyzed for the background. The volatiles were collected by using a dynamic headspace sampling system and solid-phase microextraction (Aharoni et al., 2003) in a glass container with 200 mg Tenax-TA resin (60/80 mesh; Supelco, Bellefont, PA, United States) packed into a plastic tube. The headspace sampling apparatus inserted a silicone tube to connect to the solid phase extraction columns and another silicone tube connected the column with air pumps at 350mmHg collecting. The collected compounds were analyzed by GC-MS (QP2010, SHIMADXU, Shimadzu Co., Tokyo). An INNOWAX column (60 cm, 0.3 mm, 0.25 μm phase thickness) was used, and the oven was programmed from 40 to 230°C (held for 5 min) at 5°C/min increments. The pressure of the helium inlet was set at 75.2 kPa with 34.6 cm/s linear velocity (split flow 8.3 μl/min). The injector temperature was kept at 240°C with the injected volume set to 1 μl and the electron energy to 70 eV. Mass spectra and reconstructed chromatograms were obtained by automatic scanning of the samples in the mass range m/z 20–500 Da. Peaks on mass chromatograms with characteristic fragments were checked for homogeneity. The identities of all compounds were determined by comparing retention times and mass fragmentation patterns with the NIST98 database (US Environmental Protection Agency, 1998) and NIST05 database (SHIMADXU, Shimadzu Co., Tokyo). For quantitative analysis, 10 μg/ml ethyl myristate was used as an internal standard.

### Protoplast Isolation

Protoplasts of floral buds were isolated from *Phalaenopsis aphrodite* as described in the study by Shen et al. (2017). Briefly, 10 g of 2-cm flower buds was sterilized in 70% alcohol for 1 min, followed by three washes in sterilized distilled water. The floral bud was cut into 1-mm^2^ pieces and immersed in sterilized water for 1 h. Then, floral buds were transferred to 10 ml enzyme solution (1 ml of 0.2M MES, 0.4 g cellulose RS, 0.2 g macerozyme R-10, 6 ml of 1M sucrose, 100 μl of 2M KCl, 50 μl of 2M CaCl_2_, 10% BSA) for incubation for 4 h on a shaker (30 rpm) in darkness at room temperature. After incubation, the enzyme–protoplast mixture was passed through steel screens of 100- and 50-μm pore sizes to remove undigested materials. The sample was centrifuged at 700 rpm for 5–10 min. The protoplasts on the surface were collected, resuspended in the wash buffer (cell and protoplast washing salt containing 0.6 M mannitol), and centrifuged at 700 rpm for 5 min; then the supernatant was discarded. After three washes, the pellet was resuspended in 0.5 ml of W5 solution (150 mM NaCl, 125 mM CaCl_2_, 5 mM KCl, 2 mM MES, pH 5.7, 5 mM glucose). Then, the supernatant was spun, removed, and resuspended in 500 μl of MMg solution (4 mM MES, pH 5.7, 0.6M mannitol, 15 mM MgCl_2_) before polyethylene glycol (PEG) 4000 treatment.

### Transient Expression of Green Fluorescent Protein Fusion Proteins

For cellular localization experiments, green fluorescent protein (GFP) was constructed either upstream or downstream from the analyzed genes, such as GFP-PbTPS3 or PbTPS3-GFP, respectively. The Gateway Cloning System (Invitrogen, Carlsbad, CA, United States) was used for generating p2FGW7-fused protein transformation constructs consisting of the open reading frame of PbTPS3 or PbTPS4 cDNA under the control of the cauliflower mosaic virus (CaMV) 35S promoter. The plasmids were transformed into protoplasts of *Phalaenopsis aphrodite* by PEG 4000 treatment. An empty vector that expresses GFP under the control of the CaMV 35S promoter served as a negative control. GFP fluorescence was observed under a Carl Zeiss LSM780 confocal laser scanner microscope (CLSM-780, Zeiss, Germany) with GFP excited at 488 nm with a krypton/argon laser and chlorophyll autofluorescence excited at 514 nm with a HeNe-Laser. The emissions were collected through a 506- to 530-nm band pass filter (for GFP) and a 680- to 750-nm band pass filter (for chloroplasts). To strengthen the image visibility and resolution of the original images, all images were enhanced to 50% sharpness and 30% contrast by using the built-in chart format of PowerPoint.

## Results

### Phylogenetic Analysis of Upregulated *TPSs* in *Phalaenopsis bellina* During Floral Development

**FIGURE 1:**
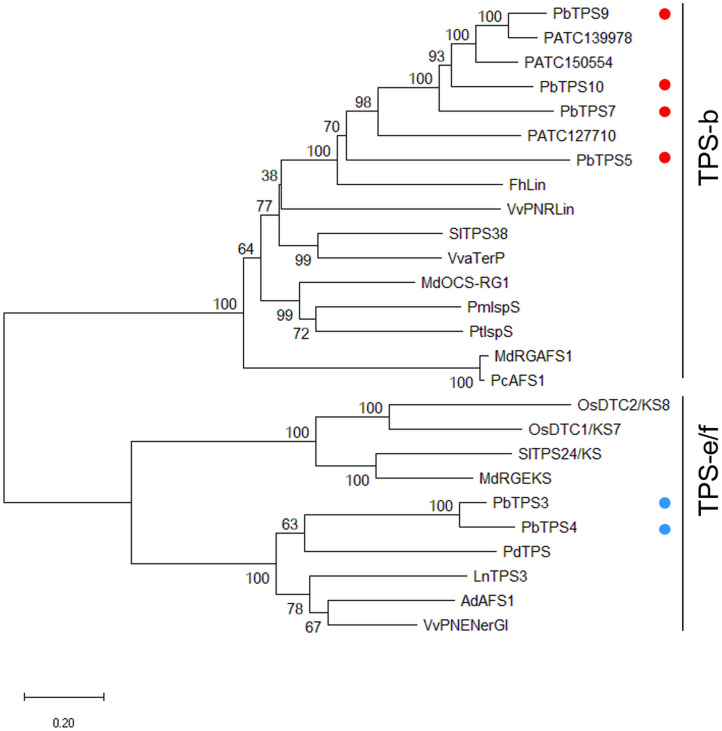
Phylogenetic analysis of *PbTPS3*, *PbTPS4*, *PbTPS5*, *PbTPS7*, *PbTPS9*, *PbTPS10*, and other plant TPSs. The coding sequence was used to construct a phylogenetic tree using the nearest neighbor-joining method with the Jones–Taylor–Thornton model and pairwise deletion with 1,000 bootstrap replicates by MEGA 7.0. The numbers at each node represent the bootstrap values. The MTPS of *Phalaenopsis* orchid are marked with red dots. *PbTPS5*, *PbTPS7*, *PbTPS9*, and *PbTPS10* are grouped in the *TPS-b* subfamily and labeled in red dots. *PbTPS3* and *PbTPS4* are grouped in the *TPS-e/f* subfamily and labeled in blue dots. Information on *TPS* genes used for phylogenetic analysis is in Supplementary Table 1.

Comparative transcriptomic analysis of scented *P. bellina* and scentless *Phalaenopsis aphrodite* revealed the upregulation of several *TPS* genes in *P. bellina* (Chuang et al., 2018b; Supplementary Figure 1B). Among them, *PbTPS5-1*, *PbTPS5-2*, *PbTPS9-1*, *PbTPS9-2-1*, *PbTPS9-2-2*, *PbTPS10-1*, *PbTPS10-2*, and *PbTPS7* are in the *TPS-b* subfamily, and *PbTPS4*, *PbTPS3-1*, and *PbTPS3-2* are in the *TPS-e/f* subfamily. Alternative splicing had occurred and produced different transcripts from the same gene, such as *TPS5-2*, which has an intron retention between exons 5 and 6 that caused a stop codon and early termination. A similar situation occurred for *TPS10-1*, in that an intron retention between exons 4 and 5 caused a stop codon and early termination (Supplementary Figure 3). So, we focused on the major transcripts for phylogenetic analysis (Supplementary Figure 1A). These were six genes: *PbTPS3*, *PbTPS4*, *PbTPS5*, *PbTPS7*, *PbTPS9*, and *PbTPS10.* Further phylogenetic analysis with known *TPS* genes of other plants showed that *PbTPS5*, *PbTPS7*, *PbTPS9*, and *PbTPS10* in the *TPS-b* subfamily (red dots, Figure 1) and *PbTPS3* and *PbTPS4* in the *TPS-e/f* subfamily (blue dots, Figure 1 and Supplementary Figure 4).

### Differential Gene Expression of *PbTPSs* Between *Phalaenopsis bellina* and *Phalaenopsis aphrodite*

**FIGURE 2:**
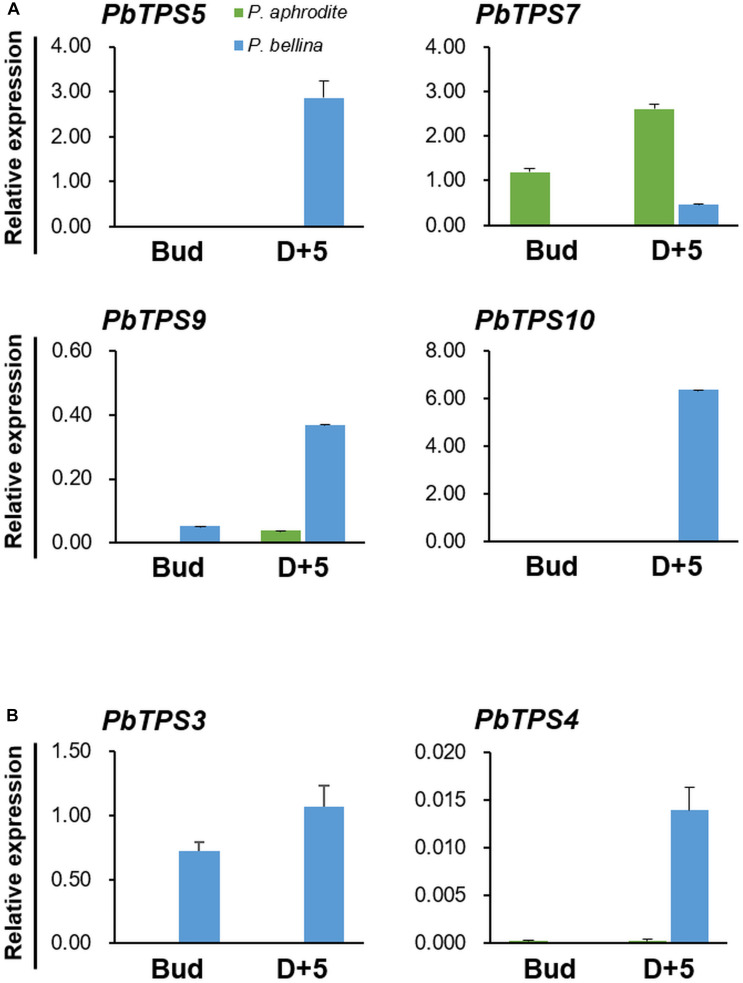
Differential gene expression of *PbTPSs* between *P. bellina* and *Phalaenopsis aphrodite*. Panels **(A,B)** are the gene expression of *TPS-b* and *TPS-e/f* members, respectively. The expression of *PbTPS*s was analyzed at the floral bud stage (Bud) and 5 days after anthesis (D + 5). Green and blue column represent the expression of *PbTPS*s in *Phalaenopsis aphrodite* and *P. bellina*, respectively. Data were obtained from three independent experiments. The expression of all genes was normalized to the reference gene, *PbActin1* (Chuang et al., 2018b). Calculation of mean and standard error was based on triplicate repeats.

To further confirm the differential expression of these six *PbTPS* genes between scented and scentless *Phalaenopsis* orchids, qRT-PCR was performed. We examined the expression of four *TPS-b* genes (*PbTPS5*, *PbTPS7*, *PbTPS9*, and *PbTPS10*) and two *TPS-e/f* genes (*PbTPS3* and *PbTPS4*) at the flower bud stage and D + 5 (Figure 2). Previously, we found that the emission of monoterpenes in *P. bellina* peaked at the full-bloom stages (D + 4 and D + 5) but was absent in floral buds (Chuang et al., 2018b). All *PbTPS* genes in *P. bellina* showed higher expression at D + 5 than at the bud stage. Except for *PbTPS7*, the qRT-PCR results were consistent with transcriptomic analysis showing an enhanced expression in the scented orchids. Expression was higher for *PbTPS3*, *PbTPS4*, *PbTPS5*, *PbTPS9*, and *PbTPS10* in *P. bellina* than in *Phalaenopsis aphrodite* in floral buds and at D + 5 (Figure 2). These results suggested that *PbTPS3*, *PbTPS4*, *PbTPS5*, *PbTPS9*, and *PbTPS10* were highly associated with floral scent monoterpene biosynthesis; *PbTPS7* was removed from the following analysis.

### Temporal and Spatial Gene Expression of the *PbTPS-b* Subfamily

**FIGURE 3:**
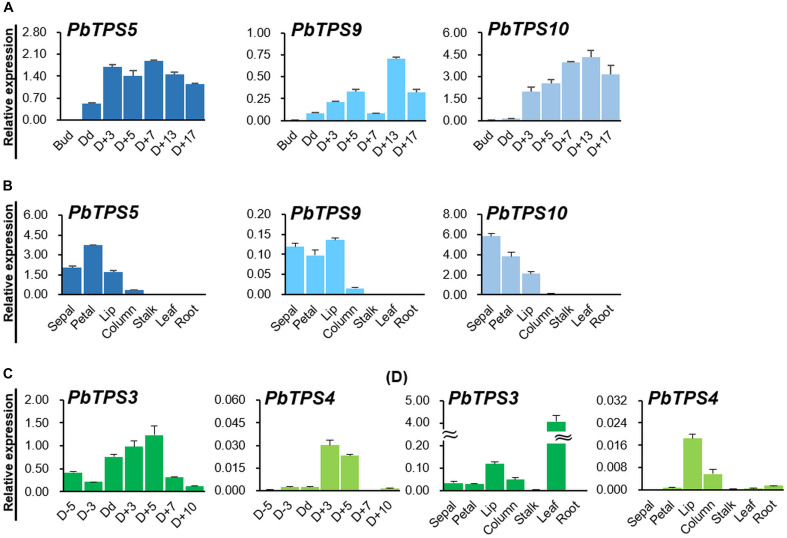
Temporal and spatial gene expression of *TPS-b* and *TPS-e/f* members *in P. bellina*. **(A)** Temporal and **(B)** spatial expression profiles of *PbTPS5*, *PbTPS9*, and *PbTPS10.* **(C)** Temporal and **(D)** spatial expression of *PbTPS3* and *PbTPS4.* Temporal expression of *PbTPS5*, *PbTPS9*, and *PbTPS10* was analyzed at floral bud stage (Bud), day of anthesis (Dd), and 3–17 days after anthesis (D + 3 to D17). Temporal expression of *PbTPS3* and *PbTPS4* was analyzed from 3 to 5 days before anthesis (D-5 and D-3) and day of anthesis (Dd) to 10 days after anthesis (D + 10) in *P. bellina*. Spatial gene expression of *PbTPS* genes was determined in various tissues, including sepal, petal, lip, column, stalk, leaf, and root. Data were obtained from three independent experiments. The expression of all genes was normalized to the reference gene, *PbActin1* (Chuang et al., 2018b). Calculation of mean and standard error was based on triplicate repeats.

We examined the temporal expression of three *TPS-b* genes from the flower bud stage to D + 17 of *P. bellina* by qRT-PCR (Figure 3). Both *PbTPS5* and *PbTPS10* showed much higher expression than *PbTPS9* in *P. bellina* during floral development (Figure 3A). *PbTPS5* and *PbTPS10* expressed highly on blooming day (Dd) and D + 3, respectively (Figure 3A). *PbTPS9* showed a biphasic pattern, with expression high on D + 5, decreased on D + 7, and then high again on D + 13 (Figure 3A).

The three *TPS-b* genes were exclusively expressed in reproductive organs, with differential profiles. *PbTPS5*, *PbTPS9*, and *PbTPS10* expressed in sepal, petal, and lip and to a lesser extent in column (Figure 3B). Especially, *PbTPS5* expressed highly in petal, whereas *PbTPS10* expressed highly in sepal.

### Temporal and Spatial Expression Profiles of the *PbTPS-e/f* Subfamily

We analyzed the temporal expression of two *PbTPS-e/f* genes, *PbTPS3* and *PbTPS4*, by qPCR from 5 days before anthesis (D − 5) to D + 10. *PbTPS3* showed increasingly high expression from Dd to D + 5, then reduced expression on D + 7, whereas *PbTPS4* showed high expression on D + 3 and D + 5, then diminished expression (Figure 3C). The expression was much lower for *PbTPS4* than for *PbTPS3* in flowers (Figure 3C). *PbTPS3* expressed mainly in the leaf, and in the flower, it expressed higher in the lip than in the sepal, petal, or column (Figure 3D). *PbTPS4* also showed high expression in the lip and column and very low expression in the sepal and petal; it was expressed in the root and leaf but at a lower level than in the floral organs (Figure 3D).

### Transient Overexpression of *PbTPS5* and *PbTPS10* in *Nicotiana tabacum*

The temporal expression profiles of *PbTPS5* and *PbTPS10* were concomitant with the emission of monoterpenes such as linalool and geraniol, so we studied their enzyme functions by ectopic overexpression in *N. tabacum.* Several compounds were produced when overexpressing *PbGDPS* to provide GDP as the substrate. A monoterpene myrcene derivative, tetrahydromyrcenol, was produced when transiently overexpressing *PbTPS10* or *PbGDPS* + *PbTPS10* in *N. tabacum* (Supplementary Figure 5). However, neither linalool nor geraniol was produced in *N. tabacum* overexpressing *PbTPS5* or *PbTPS10. N. tabacum* may have no other orchid-related genes required for monoterpene biosynthesis and hinder the production of linalool or geraniol.

### Transient Overexpression of *PbTPS5* and *PbTPS10* in Scentless *Phalaenopsis*

We attempted the transient overexpression of *PbTPS5* and *PbTPS10* in the scentless species *Phalaenopsis aphrodite* and a scentless cultivar V3. Overexpression of *PbTPS5* and *PbTPS10* with or without *PbGDPS* in *Phalaenopsis aphrodite* did not induce the terpene production in the emitted volatiles or the internal non-volatiles (Supplementary Figures 6A,C), even though monoterpenes of α-terpineol and α-cedrene were detected in the non-volatile compounds in the overexpression of *PbGDPS* in *Phalaenopsis aphrodite* This observation suggests that other enzymes or factors might be needed to produce volatile monoterpenes in *Phalaenopsis aphrodite*. Further overexpression of *PbTPS5* and *PbTPS10* was performed in V3, and the production of tetrahydromyrcenol and sesquiterpenoids was enhanced, but neither linalool nor geraniol was detected in the volatiles and non-volatiles of V3 (Supplementary Figures 6B,D). Previously, we showed that the scentless *Phalaenopsis* orchids lack *PbbZIP4* and *PbbHLH4* transcription factors for floral volatile biosynthesis (Chuang et al., 2018a, b). Hence, some other factors (such as transcription factors) may be required and are missing in the scentless orchid flowers. Alternatively, the presence of repressors may hinder the monoterpene biosynthesis in the scentless *Phalaenopsis* orchids, even though the key enzymes are provided.

### Transient Overexpression of *PbTPS5* and *PbTPS10* in a Scented *Phalaenopsis* Cultivar

**FIGURE 4:**
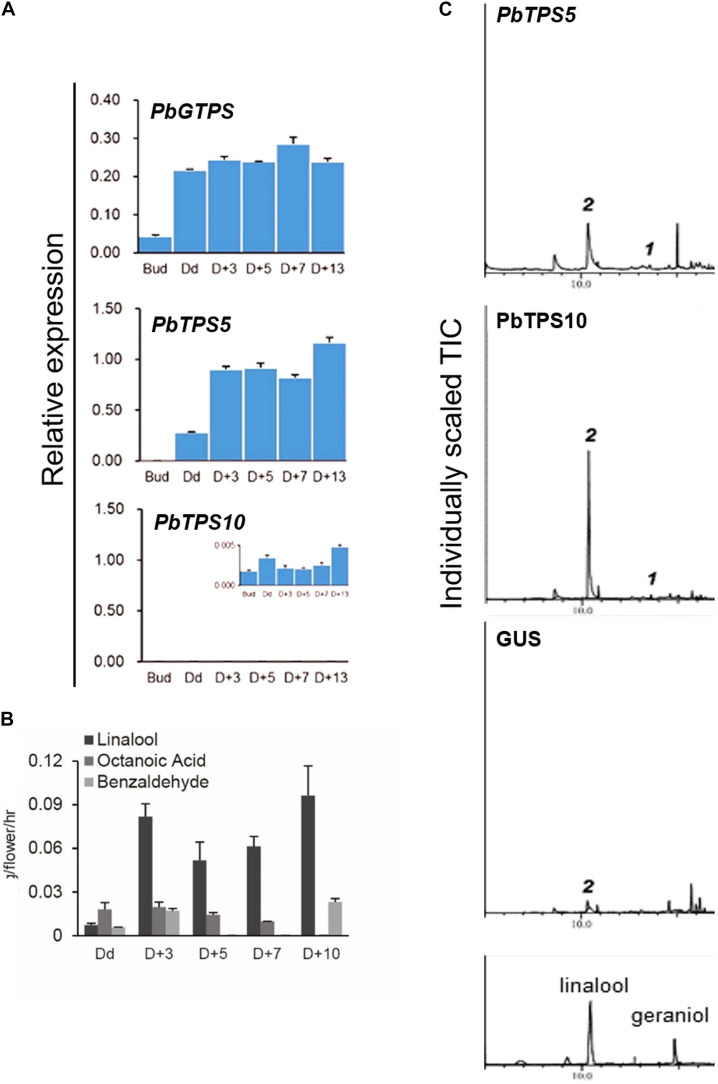
**(A)** qRT-PCR analysis of gene expression of *PbGDPS*, *PbTPS5*, and *PbTPS10* in flowers. The inset for *PbTPS10* is the enlargement of the original scale. **(B)** Floral volatile organic compounds (VOCs) emitted from *P.* I-Hsin Venus. **(C)** Functional characterization of *PbTPS5* and *PbTPS10* transiently overexpressed in Venus. Plants overexpressing GUS were a negative control.

We then overexpressed *PbTPS5* and *PbTPS10* in a scented cultivar, Venus, an offspring of *P. bellina* that has gone through more than 10 generations of crossing. In contrast to *P. bellina* producing one or two flowers and with strong scent, Venus is floriferous (Supplementary Figure 2B) but with reduced floral scent. Before the overexpression, we analyzed the endogenous gene expression and floral scent emission of Venus. We first detected the scent compounds of Venus and examined the gene expression profiles of endogenous *PbGDPS*, *PbTPS5*, and *PbTPS10.* Venus produced VOCs of linalool, octanoic acid, and benzaldehyde in flowers, with linalool as the major floral scent, yet no geraniol was detected (Figure 4B). The floral scent emission followed a similar pattern as that of *P. bellina* (i.e., the strong scent was detected from D + 3). Concomitantly, *PbGDPS* and *PbTPS5* significantly expressed from Dd and D + 3, respectively (Figure 4A), yet with little or no expression of *PbTPS10* during floral development from flower bud to D + 13 (Figure 4A). This result implied that *PbTPS5* alone can account for linalool biosynthesis.

Cauliflower mosaic virus 35S promoter was used to drive the overexpression of both *PbTPS5* and *PbTPS10*. The emission of linalool was enhanced with transient overexpression of *PbTPS5* and *PbTPS10* in Venus floral tepals (peak 2, Figure 4C). Thus, both *PbTPS5* and *PbTPS10* caused a 7.7- and 29.0-fold increase, respectively, of linalool production as compared with GUS. In addition, emission of tetrahydromyrcenol was detected overexpression of *PbTPS5* and *PbTPS10*, respectively (peak 1, Figure 4C). However, geraniol was not produced on overexpressing *PbTPS5* or *PbTPS10* in Venus.

### Functional Characterization With Purified *TPS-b* Recombinant Proteins Ectopically Overexpressed in *Escherichia coli*

**FIGURE 5:**
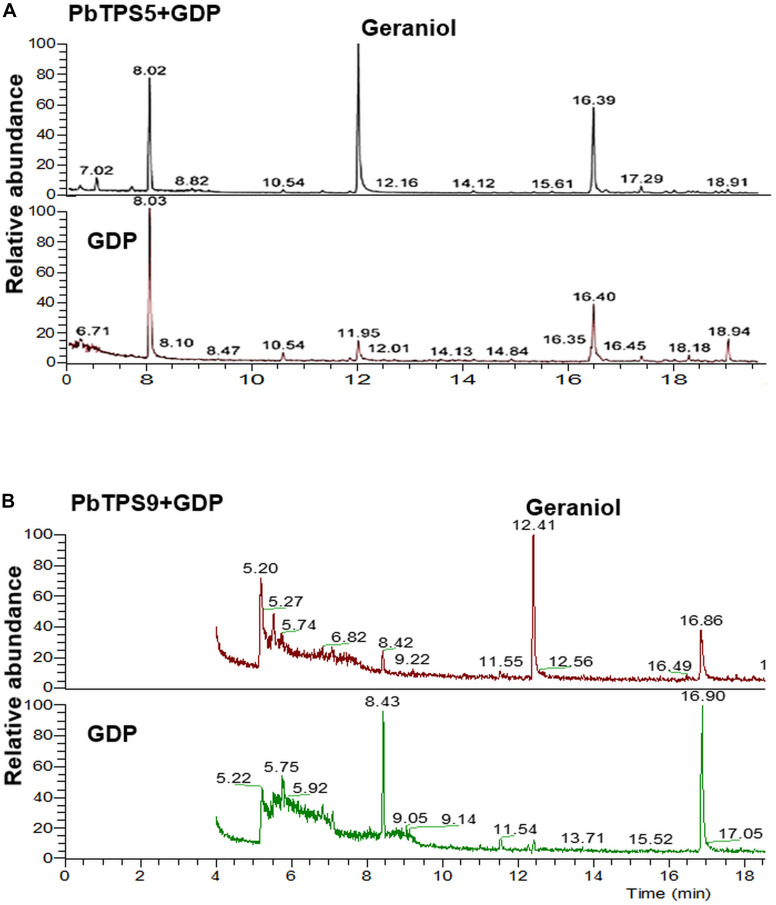
Functional characterization of purified recombinant protein of **(A)** PbTPS5 + GDP and **(B)** PbTP9 + GDP. GC-MS analysis of authentic linalool standard incubated with enzyme assay buffer. As negative controls, reactions were performed with only GDP added without any recombinant proteins.

To further confirm the enzyme activity, *in vitro* assay was performed with purified recombinant proteins ectopically expressed in *E. coli. PbTPS5* was cloned into PET-28b with 6x His at the N- and C-terminus and transformed into *E. coli*. C41 (DE3). The affinity-purified PbTPS5 protein was assayed in a buffer containing substrate GDP, and the products were analyzed by GC-MS. Geraniol was detected as the main product catalyzed by PbTPS5 (Figure 5A).

The enzyme activity of PbTPS9 was examined with the purified recombinant proteins as described above. The product of PbTPS9 was geraniol (Figure 5B).

### Functional Characterization of Purified *TPS-e/f* Recombinant Proteins Ectopically Overexpressed in *Escherichia coli*

**FIGURE 6:**
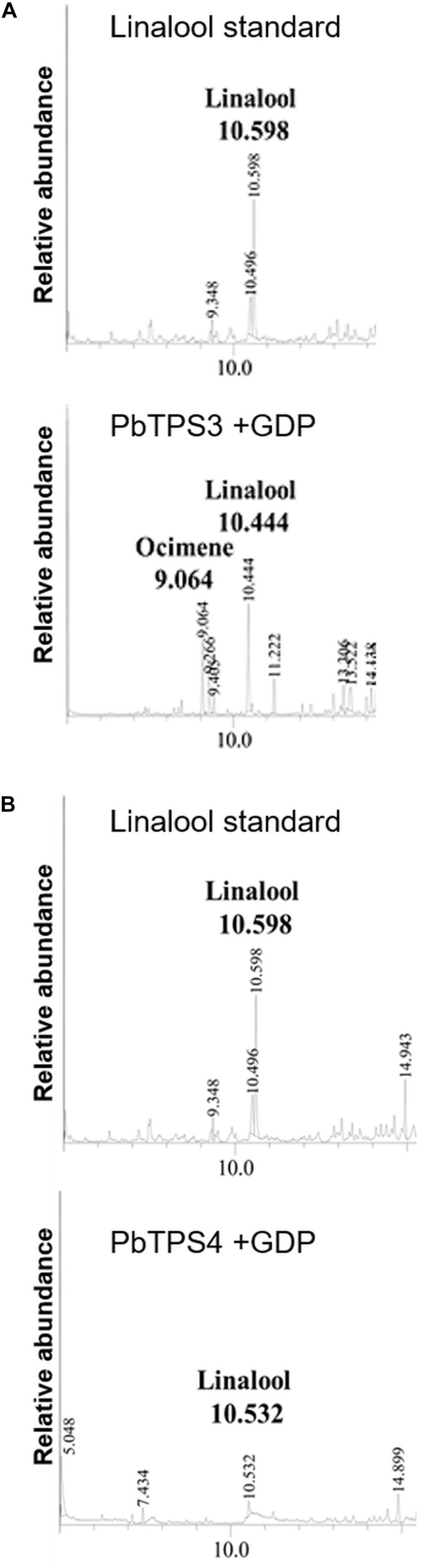
Functional characterization of purified recombinant protein of **(A)** PbTPS3 + GDP and **(B)** PbTPS4 + GDP. GC-MS analysis of authentic linalool standard incubated with enzyme assay buffer.

To functionally characterize the two *TPS-e/f* genes PbTPS3 and PbTPS4, we analyzed the affinity-purified recombinant proteins by using three different prenyl diphosphates as substrates, namely, GDP (C10), FDP (C15), and GGDP (C20). The reaction products were analyzed by GC-MS. PbTPS3 was a dual enzyme as a linalool/(β)-cis-ocimene synthase (Figure 6A). The authentic standard of linalool was used for comparing retention time. PbTPS4 was a linalool synthase and used GDP as the substrate (Figure 6B). Neither FDP nor GGDP could be catalyzed by PbTPS3 and PbTPS4 to produce sesquiterpene or diterpene (Supplementary Figure 7). Overall, these data indicate that PbTPS3 and PbTPS4 are the MTPS that exclusively produced linalool and (β)-cis-ocimene, or linalool only, respectively.

### Subcellular Localization of PbTPS3 and PbTPS4

Monoterpene synthases are mainly present in the plastids. However, both PbTPS3 and PbTPS4 lack typical transit peptides for chloroplast localization (Supplementary Figure 8). We then performed the prediction of the subcellular localizations according to their amino acid sequences with various software packages. Unexpectedly, differential prediction results were obtained. For example, ChloroP 1.1 predicted that both PbTPS3 and PbTPS4 were not localized in the chloroplasts. TargetP suggested that they were localized in other sites and mitochondria, respectively. pSORT II indicated the possible localization of PbTPS3 in the cytosol (69.6%) and nucleus (26.1%) and possible localization of PbTPS4 in the nucleus (34.8%) and cytosol and mitochondria (21.7%). Wolf PSORT indicated that PbTPS3 and PbTPS4 possibly localized in the nucleus and chloroplast, respectively.

**FIGURE 7:**
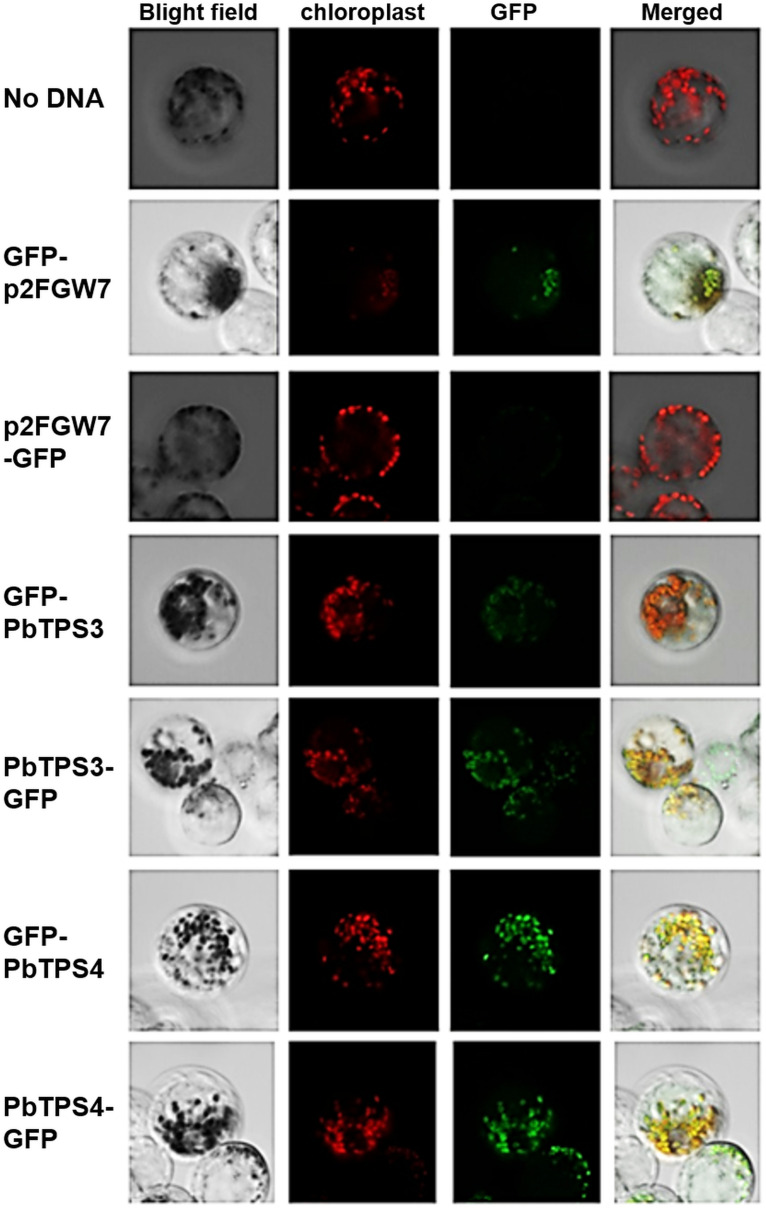
Subcellular localization of *PbTPS3* and *PbTPS4*. Protoplasts of *Phalaenopsis aphrodite* tepals were transformed with GFP fusion proteins (GFP-PbTPS3, PbTPS3-GFP, GFP-PbTPS4, and PbTPS4-GFP). Images were taken by confocal laser scanning microscopy. Bright field, the image in bright field; Chloroplast with autofluorescence image false-colored in red; GFP, GFP fluorescence image false-colored in green; Merge, merge of bright field, chloroplast and GFP fluorescence images.

Therefore, subcellular localization of PbTPS3 and PbTPS4 was determined with GFP added at the N- or C-terminus of the full-length cDNAs (Figure 7). These constructs were transformed into protoplasts isolated from mature flower buds of *Phalaenopsis aphrodite*, and the GFP signal and autofluorescence emitted from chloroplasts were examined by confocal laser scanning microscopy. The GFP fluorescence of both PbTPS3 and PbTPS4 fusion proteins was exclusively overlaid with the autofluorescence signal of chloroplasts, which suggests that they are localized exclusively in the chloroplast (Figure 7). In contrast, the controls of no DNA added or empty vector showed no or very low GFP signal, respectively (Figure 7). These results confirmed that both PbTPS3 and PbTPS4 were localized in the plastids even without a typical transit peptide signal.

## Discussion

### Both *TPS-b* and *TPS-e/f* Subfamilies Are Involved in the Biosynthesis of Monoterpenes in *Phalaenopsis bellina* Flower

**FIGURE 8:**
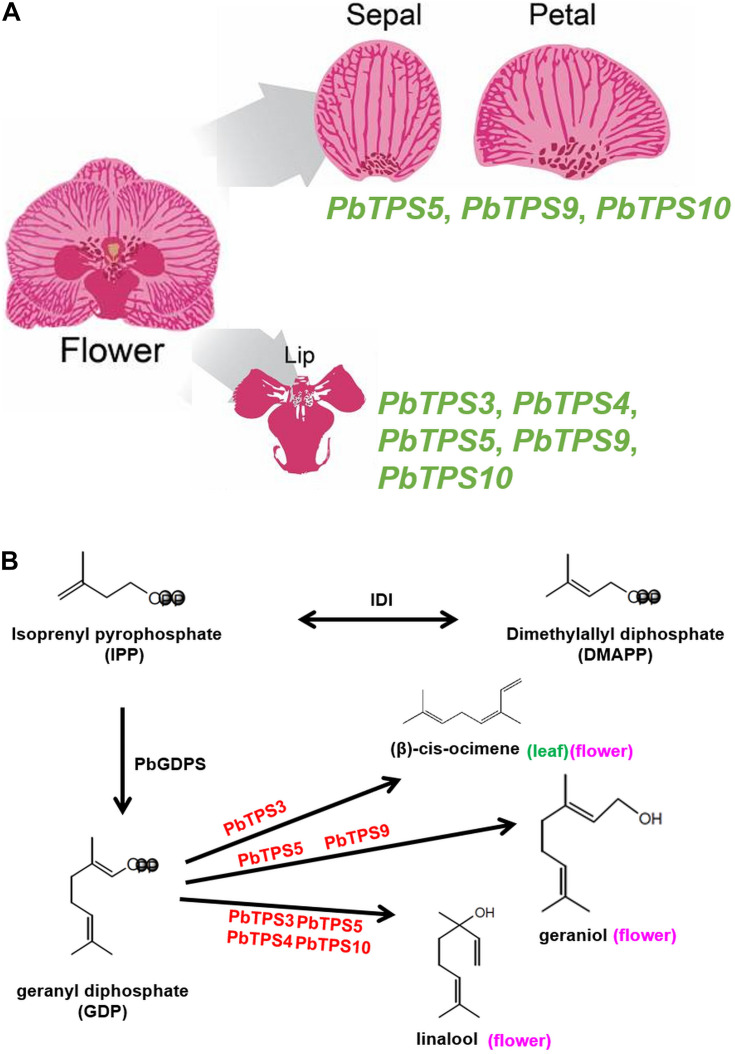
**(A)** The biosynthesis pathway of linalool, geraniol, and (β)-*cis*-ocimene in flower or (β)-*cis*-ocimene in the leaf of *P. bellina* from GDP. More than one enzyme can use GDP as substrate for producing linalool (*PbTPS3*, *PbTPS4*, *PbTPS5*, and *PbTPS10*), geraniol (*PbTPS5* and *PbTPS9*), or (β)-*cis*-ocimene (*PbTPS3*). In addition, one enzyme may produce more than one product using GDP as the substrate, such as *PbTPS3* for both linalool and (β)-*cis*-ocimene biosynthesis. **(B)** Various *PbTPS* genes involved in monoterpene biosynthesis in a single flower.

In addition to the three *TPS* genes in the *TPS-b* subfamily with an enhanced expression in *P. bellina* as compared with *Phalaenopsis aphrodite*, two *TPS* genes in the *TPS-e/f* subfamily were also enriched. In *P. bellina*, differential gene expression of *PbTPS* genes is involved in floral monoterpene biosynthesis in various parts of a single flower. For example, *PbTPS5*, *PbTPS9*, and *PbTPS10* were involved in monoterpene biosynthesis in tepal (sepal and petal) and *PbTPS3*, *PbTPS4*, *PbTPS5*, *PbTPS9*, and *PbTPS10* in lip (Figure 8A). In addition, multiple *TPS* genes are involved in the same enzymatic function, and one *TPS* gene may catalyze more than one product of monoterpenes. For example, PbTPS3, PbTPS4, PbTPS5, and PbTPS10 produce linalool, and PbTPS5 and PbTPS9 produce geraniol. In addition, PbTPS3 produces (β)-cis-ocimene, with *PbTPS3* expressed in both flower and leaf. PbTPS3 has a dual enzyme activity of linalool synthase/(β)-cis-ocimene synthase (Figure 8B).

Both PbTPS3 and PbTPS4 contain a DDXXD and NSE/DTE motif for GDP binding, and they lack the RRX8W motif for cyclic monoterpene synthesis (Supplementary Figure 8). PbTPS3 is the dual function enzyme for producing acyclic monoterpenes, including linalool and (β)-cis-ocimene. It was expressed in flower and to a much higher extent in leaf (Figure 3D). However, GC-MS analysis of leaf VOCs revealed only 1% (β)-cis-ocimene but no linalool (Supplementary Figure 9). The heterologous expression of geraniol synthase from *Ocimum basilicum* in *E. coli* and plant systems revealed that heterologous expression affects the amount of geraniol production in the leaf tissue or *E. coli* (Fischer et al., 2013). Therefore, the enzymatic functions of *TPS* genes depend on the enzyme amino acid sequence or substrate and also the conditions of an enzymatic reaction, especially when comparing plant and bacterial systems (Brillada et al., 2013).

### Functional Characterization of *PbTPS5* and *PbTPS10*

*PbTPS5* showed the enzyme activity of linalool synthase when overexpressed in Venus, and Venus itself produced only linalool and expressed *PbTPS5* slightly (Figure 3). Negative regulators of *PbTPS10* may be present in the Venus flowers and prevent its gene expression and production of linalool. Previously, differences in biochemical conditions between plant and microbial expression systems produce different products (Kollner et al., 2004). These differences between heterologous systems could result from the protein conformational change in the binding pocket due to different environments (e.g., pH, ion concentrations) or different posttranslational modifications (Fischer et al., 2013). For monoterpene synthesis, GDP is the precursor, and the conversion of GDP into monoterpenols involves the formation of a carbocation intermediate (Ilc et al., 2016). Any subtle differences in protein conformation altered by the environment (chemicals or other proteins) could affect the profile of carbocation intermediate, thus producing different products (Fischer et al., 2013). In addition, the presence of other enzymes that convert one monoterpenol into another monoterpenol cannot be ruled out because of the many enzymes in plants. The other factor that can be taken account in affecting the production of monoterpenes is intracellular pH. Changes in intracellular pH can cause the chemical transformation of monoterpenols. Previous study found that monoterpenols tended to interconvert under acidic condition but are stable at neutral pH (Fischer et al., 2011). Previously, we detected the pH value of various colored *Phalaenopsis* orchids and found that it increases from red-purple, white, purple-violet to violet blue color from pH 4.77 to 5.54 (Liang et al., 2020). Although the white flowers of *Phalaenopsis aphrodite* and V3 have similar pH values (5.05 and 5.13, respectively), different compounds were produced by overexpressing TPS genes in these two orchids, which suggests that the pH value was not the major effect on the content of volatiles in *Phalaenopsis* orchids (Supplementary Figure 5).

However, *in vitro* enzyme activity assay revealed that geraniol was the main product catalyzed by purified recombinant *PbTPS5*. With overexpression of *PbTPS5* and *PbTPS10* in Venus, linalool was the only product for both enzymes, with *PbTPS10* displaying high ability for linalool production. In addition, molecular modeling and docking were performed by using SwissDock program with both *PbTPS5* and *PbTPS10* as targets and GDP as a ligand. The ligand was difficult to dock into the active site of *PbTPS5* as compared with *PbTPS10*, which implies that the binding pocket was easier to access with *PbTPS10* than with *PbTPS5*. This finding explains why the activity of *PbTPS10* was better than that of *PbTPS5* in monoterpene synthesis. Within the flower of *P. bellina*, *PbTPS10* mainly expressed in the sepal and slightly in the petal, whereas *PbTPS5 is* mainly expressed in the petal but less in the sepal. When combining the gene expression and enzyme activity data, the monoterpene biosynthesis in different floral organs of *P. bellina* could be driven by different genes and for different scent compounds. Recent reports showed that many TPS genes can produce more than one terpenoid, which could be related to the size of the active cavity. Thus, the amino acid sequences of *TPS* genes are among the factors that affect the TPS functional property (Pazouki and Niinemets, 2016). Also, previous *TPS* studies indicated that the outcome of *in planta* experiments could be affected by the plants chosen; for instance, when geraniol synthase from *O. basilicum* was expressed in grapevine, citronellol and nerol were produced (Arendt et al., 2016). The products of *TPS* genes may vary in different host plants because the function of MTPS is based on not only the amino acid sequence but also the cellular background (Fischer et al., 2013).

### Differential Positive and Negative Gene Regulation of *PbTPS5* and *PbTPS10*

Even though the coding sequences of PbTPS5 and PbTPS10 are similar, they showed distinct temporal and spatial expression profiles. For example, the temporal expression of *PbTPS5* started on the blooming date, whereas that of *PbTPS10* started on D + 3. Spatially, *PbTPS5* expressed highly in the petal, and *PbTPS10* expressed highly in the sepal, and both expressed similarly in the lip. In addition, these two genes revealed distinct profiles of transcriptional regulation (Chuang et al., 2018b). Both *PbbZIP4* and *PbNAC1* positively regulated the promoter of *PbTPS5* but did not affect the promoter of *PbTPS10* (Chuang et al., 2018b). Conversely, both *PbbHLH4* and *PbERF9* highly positively regulated the promoter of *PbTPS10* but did not affect that of *PbTPS5*. In contrast, *PbbHLH2* and *PbbZIP26* negatively regulated *PbTPS5*, and *PbbHLH5*, *PbbZIP26*, and *PbMYB22* negatively regulated *PbTPS10* (Chuang et al., 2018b). Even though both *PbTPS5* and *PbTPS10* were negatively regulated by *PbbZIP26*, only *PbTPS10* expression was inhibited in Venus, which suggests that both bHLH5 and MYB22 may function as negative regulators to prevent the expression of *PbTPS10.* MYB transcription factor has a negative regulation role in floral scent biosynthesis (Reddy et al., 2017). Further assessments will be needed to elucidate the negative regulation of floral scent biosynthesis.

### Functional Characterization of *PbTPS3* and *PbTPS4*

The purified recombinant *PbTPS3* had dual enzyme functions as a linalool/(β)-cis-ocimene synthase, whereas that of the purified recombinant *PbTPS4* produced only linalool. Both *PbTPS3* and *PbTPS4* were expressed in reproductive organs, including sepal, petal, and lip. In addition, *PbTPS3* was expressed in the column at a similar level as in the sepal, whereas *PbTPS4* was expressed much lower in the column. So, the major floral scent of *P. bellina* in the flower tepal (sepal and petal) is dominated by linalool and geraniol, whereas the lip has the floral scent of (β)-cis-ocimene in addition to linalool, and the column has the floral scent of (β)-cis-ocimene. Emission of (β)-cis-ocimene is 15.8 ng/flower/h, accounting for 2% of the floral scent of *P. bellina* (Chuang et al., 2018b).

### *PbTPS3* and *PbTPS4* Are New Members of the *TPS-e/f* Subfamily

*TPS* enzymes in the *TPS-e/f* subfamily have dual functions or play different roles. For example, the (β)-cis-ocimene/farnesene synthase of *Actinidia deliciosa* has dual functions, and it produces the (β)-cis-ocimene and farnesene mixture for pollinator attraction (Nieuwenhuizen et al., 2009). The foregoing observations indicate that the *TPS* genes in the *TPS-e/f* subfamily have various ecological roles in plants, including floral scent biosynthesis and plant defense. For example, linalool synthase is involved in linalool biosynthesis in *Clarkia concinna* and *C. breweri*. The expression of linalool synthase in the stigma of *C. breweri* leads to the emission of linalool and linalool oxides to serve as pollinator attractants (Dudareva et al., 1996). In addition, the geranyllinalool synthase of *A. thaliana* can produce the diterpene product of geranyllinalool for plant defense (Herde et al., 2008). Thus, the (β)-cis-ocimene/linalool synthase *PbTPS3* and linalool synthase *PbTPS4* in *P. bellina* are new members in the *TPS-e/f* subfamily.

Both linalool and (β)-cis-ocimene have a defense role against insect herbivores in many plants such as cotton, soybean, and rice (Pare and Tumlinson, 1999). The promoter of *PbTPS3* contains three and 10 binding domains of the plant defense transcription factors WBOXATNPR1 and WRKY71OS, respectively, and the promoter of *PbTPS4* contains three binding domains for WRKY71OS (Supplementary Figure 10). Thus, both *PbTPS3* and *PbTPS4* may play a role in defense in *P. bellina*. *PbTPS3* may play different roles in different organs of *P. bellina*. In flower, *PbTPS3* plays a role in linalool synthesis to attract pollinators but plays a defense role in the leaf.

### The Evolution and Diversity of the *TPS-e/f* Subfamily

Linalool synthases have been identified in two *TPS* subfamilies, namely, *TPS-b* and *TPS-e/f*, so linalool synthase functionality has arisen multiple times independently during evolution (Nieuwenhuizen et al., 2009). The *TPS-e/f* subfamily is unique in that the members contain a conserved conifer diterpene internal sequence (CDIS) domain. Thus, the CDIS domain exists in a common DTPS ancestor but was lost during the evolution of MTPS and STPS (Brodmann et al., 2008). The *TPS-f* subfamily is probably dicot-specific, whereas the *TPS-e* subfamily is not (Chen et al., 2011). The *TPS-e/f* subfamily has the DDXXD conserved motif and lacks the DXDD conserved motif. From multiple alignment of amino acid sequences of the TPS-e/f subfamily in *P. equestris*, *P. bellina*, and other plants, *PbTPS3* and *PbTPS4* are cladded in the *TPS-e/f* subfamily (Figure 1; Supplementary Figures 4, 8). Our study indicates that *PbTPS3* and *PbTPS4* from orchids belong to the *TPS-e/f* subfamily and enlarge the content of this subfamily.

The subcellular localization analysis indicated that both *PbTPS3* and *PbTPS4* GFP fusion proteins are located in the plastids. The temporal expression patterns of *PbTPS3* and *PbTPS4* were concomitant with the volatile emission pattern of *P. bellina*. Taken together, *PbTPS3* and *PbTPS4* play different ecological roles of (β)-cis-ocimene/linalool and linalool biosynthesis in *P. bellina*.

## Data Availability Statement

The datasets presented in this study can be found in online repositories. The names of the repository/repositories and accession number(s) can be found in the article/Supplementary Material.

## Author Contributions

HH, Y-WK, and Y-CC performed the experiments of quantitative real-time RT-PCR, did functional characterization, and analyzed the data. Y-PY performed the original screening. L-MH performed the phylogenetic analysis. M-FJ assisted the sequence analysis and molecular docking. W-HC provided the suggestions for plant materials. H-HC conceived research plans and composed the article with assistances of all the authors, completed the writing, and served as the corresponding author for communication. All authors contributed to the article and approved the submitted version.

## Conflict of Interest

The authors declare that the research was conducted in the absence of any commercial or financial relationships that could be construed as a potential conflict of interest.

## Footnotes

1 http://www.orchideya.ca/

## References

[B1] Aharoni A. Giri A. P. Deuerlein S. Griepink F. De Kogel W. J. Verstappen F. W. (2003). Terpenoid metabolism in wild-type and transgenic *Arabidopsis* plants. *Plant Cell* 15 2866–2884. 10.1105/tpc.016253 14630967 PMC282818

[B2] Arendt P. Pollier J. Callewaert N. Goossens A. (2016). Synthetic biology for production of natural and new-to-nature terpenoids in photosynthetic organisms. *Plant J.* 87 16–37. 10.1111/tpj.13138 26867713

[B3] Bohlmann J. Meyer-Gauen G. Croteau R. (1998). Plant terpenoid synthases: molecular biology and phylogenetic analysis. *Proc. Natl. Acad. Sci. U. S. A.* 95 4126–4133. 10.1073/pnas.95.8.4126 9539701 PMC22453

[B4] Brillada C. Nishihara M. Shimoda T. Garms S. Boland W. Maffei M. E. (2013). Metabolic engineering of the C16 homoterpene TMTT in *Lotus japonicus* through overexpression of (E,E)-geranyllinalool synthase attracts generalist and specialist predators in different manners. *New Phytol.* 200 1200–1211. 10.1111/nph.12442 23952336

[B5] Brodmann J. Twele R. Francke W. Holzler G. Zhang Q. H. Ayasse M. (2008). Orchids mimic green-leaf volatiles to attract prey-hunting wasps for pollination. *Curr. Biol.* 18 740–744. 10.1016/j.cub.2008.04.040 18472423

[B6] Chen F. Tholl D. Bohlmann J. Pichersky E. (2011). The family of terpene synthases in plants: a mid-size family of genes for specialized metabolism that is highly diversified throughout the kingdom. *Plant J.* 66 212–229. 10.1111/j.1365-313X.2011.04520.x 21443633

[B7] Chen Y. H. Tsai Y. J. Huang J. Z. Chen F. C. (2005). Transcription analysis of peloric mutants of *Phalaenopsis* orchids derived from tissue culture. *Cell Res.* 15 639–657. 10.1038/sj.cr.7290334 16117854

[B8] Chuang Y. C. Hung Y. C. Hsu C. Y. Yeh C. M. Mitsuda N. Ohme-Takagi M. (2018a). A dual repeat cis-element determines expression of GERANYL DIPHOSPHATE SYNTHASE for monoterpene production in *Phalaenopsis* orchids. *Front. Plant Sci.* 9:765. 10.3389/fpls.2018.00765 29922327 PMC5996158

[B9] Chuang Y. C. Hung Y. C. Tsai W. C. Chen W. H. Chen H. H. (2018b). PbbHLH4 regulates floral monoterpene biosynthesis in *Phalaenopsis* orchids. *J. Exp. Bot.* 69 4363–4377. 10.1093/jxb/ery246 29982590 PMC6093345

[B10] Chuang Y. C. Lee M. C. Chang Y. L. Chen W. H. Chen H. H. (2017). Diurnal regulation of the floral scent emission by light and circadian rhythm in the *Phalaenopsis* orchids. *Bot. Stud.* 58:50. 10.1186/s40529-017-0204-8 29143225 PMC5688052

[B11] Colquhoun T. A. Kim J. Y. Wedde A. E. Levin L. A. Schmitt K. C. Schuurink R. C. (2011). PhMYB4 fine-tunes the floral volatile signature of *Petunia x hybrida* through PhC4H. *J. Exp. Bot.* 62 1133–1143. 10.1093/jxb/erq342 21068208 PMC3022401

[B12] Dudareva N. Cseke L. Blanc V. M. Pichersky E. (1996). Evolution of floral scent in *Clarkia*: novel patterns of S-linalool synthase gene expression in the C-breweri flower. *Plant Cell* 8 1137–1148. 10.1105/tpc.8.7.1137 8768373 PMC161191

[B13] Fischer M. J. Meyer S. Claudel P. Bergdoll M. Karst F. (2011). Metabolic engineering of monoterpene synthesis in yeast. *Biotechnol. Bioeng.* 108 1883–1892. 10.1002/bit.23129 21391209

[B14] Fischer M. J. C. Meyer S. Claudel P. Perrin M. Ginglinger J. F. Gertz C. (2013). Specificity of *Ocimum basilicum* geraniol synthase modified by its expression in different heterologous systems. *J. Biotechnol.* 163 24–29. 10.1016/j.jbiotec.2012.10.012 23108028

[B15] Herde M. Gartner K. Kollner T. G. Fode B. Boland W. Gershenzon J. (2008). Identification and regulation of TPS04/GES, an *Arabidopsis* geranyllinalool synthase catalyzing the first step in the formation of the insect-induced volatile C(16)-homoterpene TMTT. *Plant Cell* 20 1152–1168. 10.1105/tpc.106.049478 18398052 PMC2390743

[B16] Hsiao Y. Y. Jeng M. F. Tsai W. C. Chuang Y. C. Li C. Y. Wu T. S. (2008). A novel homodimeric geranyl diphosphate synthase from the orchid *Phalaenopsis bellina* lacking a DD(X)(2-4)D motif. *Plant J.* 55 719–733. 10.1111/j.1365-313X.2008.03547.x 18466308

[B17] Hsiao Y. Y. Tsai W. C. Kuoh C. S. Huang T. H. Wang H. C. Wu T. S. (2006). Comparison of transcripts in *Phalaenopsis* bellina and *Phalaenopsis equestris* (Orchidaceae) flowers to deduce monoterpene biosynthesis pathway. *BMC Plant Biol.* 6:14. 10.1186/1471-2229-6-14 16836766 PMC1540424

[B18] Hsieh M. H. Pan Z. J. Lai P. H. Lu H. C. Yeh H. H. Hsu C. C. (2013). Virus-induced gene silencing unravels multiple transcription factors involved in floral growth and development in *Phalaenopsis* orchids. *J. Exp. Bot.* 64 3869–3884. 10.1093/jxb/ert218 23956416 PMC3745740

[B19] Hsu C. C. Chen Y. Y. Tsai W. C. Chen W. H. Chen H. H. (2015). Three R2R3-MYB Transcription factors regulate distinct floral pigmentation patterning in *Phalaenopsis* spp. *Plant Physiol.* 168 175–191. 10.1104/pp.114.254599 25739699 PMC4424010

[B20] Ilc T. Parage C. Boachon B. Navrot N. Werck-Reichhart D. (2016). Monoterpenol oxidative metabolism: role in plant adaptation and potential applications. *Front. Plant Sci.* 7:509. 10.3389/fpls.2016.00509 27200002 PMC4844611

[B21] Kollner T. G. Schnee C. Gershenzon J. Degenhardt J. (2004). The sesquiterpene hydrocarbons of maize (Zea mays) form five groups with distinct developmental and organ-specific distribution. *Phytochemistry* 65 1895–1902. 10.1016/j.phytochem.2004.05.021 15279995

[B22] Liang C. Y. Rengasamy K. P. Huang L. M. Hsu C. C. Jeng M. F. Chen W. H. (2020). Assessment of violet-blue color formation in *Phalaenopsis* orchids. *BMC Plant Biol.* 20:212. 10.1186/s12870-020-02402-7 32397954 PMC7218627

[B23] Lichtenthaler H. K. Schwender J. Disch A. Rohmer M. (1997). Biosynthesis of isoprenoids in higher plant chloroplasts proceeds via a mevalonate-independent pathway. *FEBS Lett.* 400 271–274. 10.1016/s0014-5793(96)01404-4 9009212

[B24] Nieuwenhuizen N. J. Wang M. Y. Matich A. J. Green S. A. Chen X. Y. Yauk Y. K. (2009). Two terpene synthases are responsible for the major sesquiterpenes emitted from the flowers of kiwifruit (*Actinidia deliciosa*). *J. Exp. Bot.* 60 3203–3219. 10.1093/jxb/erp162 19516075 PMC2718223

[B25] Pare P. W. Tumlinson J. H. (1999). Plant volatiles as a defense against insect herbivores. *Plant Physiol.* 121 325–331. 10.1104/pp.121.2.325 10517823 PMC1539229

[B26] Pazouki L. Niinemets U. (2016). Multi-Substrate Terpene Synthases: their occurrence and physiological significance. *Front. Plant Sci.* 7:1019. 10.3389/fpls.2016.01019 27462341 PMC4940680

[B27] Reddy V. A. Wang Q. Dhar N. Kumar N. Venkatesh P. N. Rajan C. (2017). Spearmint R2R3-MYB transcription factor MsMYB negatively regulates monoterpene production and suppresses the expression of geranyl diphosphate synthase large subunit (MsGPPS.LSU). *Plant Biotechnol. J.* 15 1105–1119. 10.1111/pbi.12701 28160379 PMC5552485

[B28] Sanguinetti A. Buzatto C. R. Pedron M. Davies K. L. Ferreira P. M. Maldonado S. (2012). Floral features, pollination biology and breeding system of *Chloraea membranacea* Lindl. (Orchidaceae: Chloraeinae). *Ann. Bot.* 110 1607–1621. 10.1093/aob/mcs221 23071217 PMC3503500

[B29] Schiestl F. P. Ayasse M. Paulus H. F. Lofstedt C. Hansson B. S. Ibarra F. (2000). Sex pheromone mimicry in the early spider orchid (*ophrys sphegodes*): patterns of hydrocarbons as the key mechanism for pollination by sexual deception. *J. Comp. Physiol. A.* 186 567–574. 10.1007/s003590000112 10947239

[B30] Shen Y. Meng D. Mcgrouther K. Zhang J. Cheng L. (2017). Efficient isolation of *Magnolia* protoplasts and the application to subcellular localization of *MdeHSF1*. *Plant Methods* 13:44. 10.1186/s13007-017-0193-3 28546825 PMC5442663

[B31] Spitzer-Rimon B. Marhevka E. Barkai O. Marton I. Edelbaum O. Masci T. (2010). *EOBII*, a gene encoding a flower-specific regulator of phenylpropanoid volatiles’ biosynthesis in Petunia. *Plant Cell* 22 1961–1976. 10.1105/tpc.109.067280 20543029 PMC2910970

