# Supplementary material for: Terpene Synthase-b and Terpene Synthase-e/f Genes Produce Monoterpenes for Phalaenopsis bellina Floral Scent
Source: Front Plant Sci. 2021 Jul 14;12:700958. doi: 10.3389/fpls.2021.700958 (PMC8318001; doi:10.3389/fpls.2021.700958)
Supplement: Supplementary file 1 [file Data_Sheet_1.PDF]

	**Original transcriptomics data of *P. bellina* were normalized by $\log_{3.22}$ that resulted identical levels for *Actin1* between RNA-seq and microarray data**[a]					**Microarray data of *P. aphrodite***	
	Log base 3.2271087					Pa Microarray	
PbsTPS ID	PbDd[b] lnFPKM	PbD_3[c] lnFPKM	PbD_5[d] lnFPKM	PbD_7[e] lnFPKM	PaTPS	PaBud[f]	PaFlow[g]
PbTPS9-1	-1.494	2.557	2.855	2.745	PATC084494	-2.233	-8.330
PbTPS9-2-1	1.513	1.583	1.470	1.484	PATC139978	-4.187	-7.369
PbTPS9-2-2	2.166	2.555	2.093	2.300	PATC150554	-0.961	-7.210
					PATC153230	-4.332	-8.330
					PATC169593	-5.375	-8.330
					PATC208458	-1.909	-6.029
PbTPS10-1	1.632	2.926	1.519	1.821	ND		
PbTPS10-2	1.892	4.575	2.725	3.263			
PbTPS7	0.579	3.840	2.713	2.230	ND		
ND					PATC127710	-8.172	-8.330
ND					PATC154404	2.307	4.549
PbTPS5-1	4.057	4.584	4.677	4.557	PATC144448	-0.347	-5.674
PbTPS5-2	3.427	4.151	4.227	4.158			
PbTPS8-1	-0.269	1.310	0.583	0.018	PATC068781	-5.613	-6.486
PbTPS8-2	2.021	3.531	3.040	3.027	PATC187424	-6.313	-8.330
PbTPS1	2.764	3.981	3.177	3.192			
PbTPS2-1	0.273	0.245	-0.288	-1.732	PATC144727	-8.172	-8.330
ND					PATC137979	0.521	-5.221
ND					PATC043551	-8.172	-8.330
					PATC133907	-8.172	-8.330
ND					PATC155674	-8.172	-8.330
					PATC175129	-8.172	-8.330
PbTPS11-1	0.530	0.206	-0.279	0.093	PATC069392	-7.101	-5.873
PbTPS11-2	0.372	-0.111	-0.625	-0.870	PATC126134	-0.914	-0.598
PbTPS6	-0.303	-0.912	-0.685	-0.468	PATC125730	-2.741	-5.057
					PATC141250	-1.149	-1.521
					PATC152801	-8.134	-8.330
					PATC200022	-6.573	-8.330
ND					PATC127461	-8.172	-8.330
					PATC127891	-8.172	-8.330
					PATC161091	-5.438	-5.977
PbTPS4	2.281	1.738	2.447	2.107	PATC148546	-8.172	-8.330
PbTPS3-2	2.091	-0.054	2.528	2.252	PATC183449	-4.415	-6.336
PbTPS3-4	0.636	1.306	1.349	1.329			

[a]Chuang, Y.C., Hung, Y.C., Tsai, W.C., Chen, W.H., and Chen, H.H. 2018. PbbHLH4 regulates floral monoterpene biosynthesis in *Phalaenopsis* orchids. *Journal of Experimental Botany* 69: 4363-4377.

[b]The day of anthesis (Dd)

[c]3 days after anthesis (D_3)

[d]5 days after anthesis (D_5)

[e]7 days after anthesis (D_7)

[f]Large bud (Bud)

[g] Fully open flower (Flow)
