# Supplementary material for: Terpene Synthase-b and Terpene Synthase-e/f Genes Produce Monoterpenes for Phalaenopsis bellina Floral Scent
Source: Front Plant Sci. 2021 Jul 14;12:700958. doi: 10.3389/fpls.2021.700958 (PMC8318001; doi:10.3389/fpls.2021.700958)
Supplement: Supplementary file 2 [file Data_Sheet_2.pdf]

Supplementary Table S1 Information of *TPS* genes used for phylogenetic analysis in Fig. 1.

	Name	Accession number	Species
TPS-b	PATC139978	-	*Phalaenopsis aphrodite*
	PATC127710	-	*Phalaenopsis aphrodite*
	PATC150554	-	*Phalaenopsis aphrodite*
	PbTPS5	MW645242	*Phalaenopsis bellina*
	PbTPS7	MW645243	*Phalaenopsis bellina*
	PbTPS9	MW645244	*Phalaenopsis bellina*
	PbTPS10	MW645245	*Phalaenopsis bellina*
	FhLin	AFP23421.1	*Freesia hybrid* cultivar
	VvPNRLin	ADR74209.1	*Vitis vinifera*
	MdOCS-RG1	AGB14628.1	*Malus domestica*
	SlTPS38	AEP82768.1	*Solanum lycopersicum*
	VvaTerP	NP_001268216.1	*Vitis vinifera*
	PmIspS	AAQ84170.1	*Pueraria montana* var. *lobata*
	PtIspS	AAQ16588.1	*Populus tremuloides*
	MdRGAFS1	AAX19772.1	*Malus domestica*
	PcAFS1	AAT70237.1	*Pyrus communis* L.
TPS-e/f	PbTPS3	MW645240	*Phalaenopsis bellina*
	PbTPS4	MW645241	*Phalaenopsis bellina*
	OsDTC2/KS8	XP_015617513.1	*Oryza sativa*
	OsDTC1/KS7	BAC56714.1	*Oryza sativa*
	SITPS24/KS	AEP82778.1	*Solanum lycopersicum*
	MdRGEKS	AFG18184.1	*Malus domestica*
	LnTPS3	AKQ19359.1	*Laurus nobilis*
	AdAFS1	ACO40485.1	*Actinidia deliciosa*
	VvPNENerGl	ADR74220.1	*Vitis vinifera*
	PdTPS	XP_017696375.1	*Phoenix dactylifera*

Supplementary Table S2 Information of *TPS* genes used for phylogenetic analysis in Supplementary Fig. S1.

Subfamily	Name	Accession No.	Species	Function/Product
TPS-b	FhLin	AFP23421.1	*Freesia hybrid* cultivar	linalool
	VvPNRLin	ADR74209.1	*Vitis vinifera*	(3R)-linalool
	MdOCS-RG1	AGB14628.1	*Malus domestica*	(*E*)-β-ocimene
	SlTPS38	AEP82768.1	*Solanum lycopersicum*	α-bergamotene
	VvaTerP	NP_001268216.1	*Vitis vinifera*	(-)-alpha-terpineol
	PmIspS	AAQ84170.1	*Pueraria montana* var. *lobata*	isoprene
	PtIspS	AAQ16588.1	*Populus tremuloides*	isoprene
	MdRGAFS1	AAX19772.1	*Malus domestica*	(E,E)-a-farnesene
	PcAFS1	AAT70237.1	*Pyrus communis* L.	(E,E)-a-farnesene
TPS-g	AmMyr	AAO41727.1	*Antirrhinum majus*	myrcene
	AtTPS14	NP_176361.2	*Arabidopsis thaliana*	(3S)-linalool
	GmNES	AEE92791.1	*Glycine max*	nerol
	ZmTPS2	NP_001105854.1	*Zea mays*	linalool, (*E*)-nerolidol, and (*E*,*E*)-geranyllinalool
TPS-a	Cstps1	AAQ04608.1	*Citrus sinensis*	valencene
	MdGDS-RG1	AGB14625.1	*Malus domestica*	germacrene-D
	SlTPS12	AEP82783.1	*Solanum lycopersicum*	β-caryophyllene and α-humulene
	NtEAS	AFJ04408.1	*Nicotiana tabacum*	5-epi-aristolochene
	EhTPS	XP_010910563.1	*Elaeis guineensis*	—
TPS-c	OsCyc2	BAD42452.1	*Oryza sativa*	ent-copalyl diphosphate
	SlTPS41	AEP82765.1	*Solanum lycopersicum*	Unknown
	SrCPS	AAB87091.1	*Stevia rebaudiana*	ent-copalyl diphosphate
TPS-e/f	OsDTC2/KS8	XP_015617513.1	*Oryza sativa*	stemar-13-ene
	OsDTC1/KS7	BAC56714.1	*Oryza sativa*	ent-cassa-12,15-diene
	SlTPS24/KS	AEP82778.1	*Solanum lycopersicum*	ent-kaurene
	MdRGEKS	AFG18184.1	*Malus domestica*	ent-kaurene
	LnTPS3	AKQ19359.1	*Laurus nobilis*	geranyllinalool
	AdAFS1	ACO40485.1	*Actinidia deliciosa*	(E,E)-a-farnesene
	VvPNENerGl	ADR74220.1	*Vitis vinifera*	(*E*)-Nerolidol and (*E*,*E*)-Geranyl linalool syn
	PdTPS	XP_017696375.1	*Phoenix dactylifera*	—

Supplementary Figure legends

Supplementary Figure S1. (A) Phylogenetic analysis of the subfamilies of putative *TPS* genes with *TPS* genes identified from other species (detailed information in Supplemental Table 2). *TPS* genes from *P. bellina* are in bold black and from *P. aphrodite* in bold grey. Bootstrap values were calculated as a percentage of 1000 replicates, with values > 50 shown. The scale bar represents 0.2 substitutions per site. (B) Comparative gene expression analysis using heatmap analysis of *TPS* genes in transcriptomic data for blooming day (Dd), 3 days post-anthesis (D+3), D+5 and D+13 in *P. bellina* and microarray data for bud and flower in *P. aphrodite.* Hierarchical clustering was performed by the centroid linkage method with uncentered correlation. The heatmap and dendrogram of the clustered data were visualized by using Java TreeView (2.11) (Freudenberg et al., 2009). The color scale ranged from red (highest expression) to white to green (lowest expression).

Supplementary Figure S2. Plant morphology for (A) *P. bellina* and (B) *P.* I-Hsin Venus. *P. bellina* has strong floral scent but has one flower in the spike at one time, while *P.* I-Hsin Venus has less scent but is floriferous.

Supplementary Figure S3. An alternative-splicing model was proposed to account for the appearance of pre-stop MTPS transcripts, PbTPS5-2 and PbTPS10-1. Gene structure analysis of *PbTPS5* and *PbTPS10* showed that both genes contain one normal transcript and one pre-stop transcript, which might result from intron retention because of alternative splicing. TSS, transcription start site.

Supplementary Figure S4. Phylogenetic analysis of *Clarkia* linalool synthase (in blue dots) and *PbTPS3*, *PbTPS4* and other plants terpene synthases. Sequence analysis was performed with MEGA5 to create a tree using the nearest neighbor-joining method. The numbers at each node represent the bootstrap values. The accession numbers of sequences used are as follows: *Perilla frutescens* limonene synthase (AAG31438.1), *Gossypium arboretum* cadinene synthase (CAA65289.1), *Cucurbita maxima* ent-kaurene synthase (AAB39482.1), *Clarkia breweri* S-linalool synthase (AAC49395.1), *Gossypium hirsutum* cadinene synthase (AAC12784.1), *Citrus limon* gama-terpinene synthase (AAM53943.1), *Citrus limon* limonene synthase 2 (AAM53946.1), *Pisum sativum* copalyl diphosphate synthase (AAB58822.1), *Mentha aquatic* linalool synthase (AAL99381.1), *Abies qrandis* pinene synthase (AAB71085.1), *Abies qrandis* limonene synthase (AAB70907.1), *Mtntha piperita* farnesene synthase (AAB95209.1), *Solanum lycopersicum* germacrene C synthase (AAC39432.1), *Salvia officinalis* cineole synthase (AAC26016.1), *Salvia officinalis* bornyl diphosphate synthae (AAC26017.1), *Salvia officinalis* sabinene synthase (AAC26018.1), *Cinnamomum tenuipile* geraniol synthase (CAD29734.2), *Mentha spicata* limonene synthase (AAC37366.1), *Clarkia concinna* linalool synthase (AAD19839.1), *Clarkia breweri*

linalool synthase 2 (AAD19840.1), *Nicotiana tatbcum* aristolochene synthase (AAA19216.1), *Arabidopsis thaliana* copalyl diphosphate synthase (AAA53632.1), *Zea mays* copalyl diphosphate synthase (AAA73960.1), *Gossypium arboretum* cadinene synthase (AAD51718.1), *Artemisia annua* linalool synthase (AAF13356.1), *Abies grandis* phellandrene synthase (AAF61453.1), *Abies grandis* terpinolene synthase (AAF61454.1), *Abies grandis* limonene/pinene synthase (AAF61455.1), *Arabidopsis thaliana* geranyllinalool synthase (Q93YV0.1), *Actinidia deliciosa* ocimene/farnesene synthase (ACO40485.1), *Oryza sativa* Japonica Group linalool synthase (ACF05530.1), *Solanum lycopersicum* terpene synthase (AEP82769.1), *Solanum lycopersicum* linalool/nerolidol synthase (AEP82767.1), *Antirrhinum majus* ocimene synthase (AAO42614.1), *Antirrhinum majus* myrcene synthase (AAO41727.1), *Arabidopsis thaliana* terpene synthase 14 (NP_001185286.1), *Medicago truncatula* chloroplast terpene synthase 3 (AAV36466.1), *Glycine max* nerol synthase (AEE92791.1), *Medicago truncatula* terpenoid synthase (ABE80305.1), and *Medicago truncatula* terpenoid synthase (ABE91630.1).

Supplementary Figure S5. (A) GC-MS of *N. tabaccum* transiently overexpressing *PbTPS5* and *PbTPS10* with or without *PbGDPS*. (B) Quantification of tetrahydromyrcenol detected in various experiments. (C) The peaks labeled in (A) are as follows: 1: Tetrahydromyrcenol; 2: Nonanol; 3: Decanal; 4: Ethyl-hexanol; 5: Dodecene; 6: Decene.

Supplementary Figure S6. VOCs detected in flowers of (A) *P. aphrodite* and (B) *P.* Sogo Yukidian 'V3' overexpressing various genes, and internal non-volatile terpenoids in flowers of (C) *P. aphrodite*, and (D) *P.* Sogo Yukidian 'V3' overexpressing various genes. Different numbers show the different independent line.

Supplementary Figure S7. Functional characterization of purified recombinant proteins of *PbTPS3* and *PbTPS4* with FDP and GGDP added as the substrates. (A) PbTPS3+FDP, (B) PbTPS3+GGDP, (C) PbTPS4+FDP, and (D) PbTPS4+GGDP, and No monoterpene products were detected in any reactions.

Supplementary Figure S8. Multiple alignment of amino acid sequences of the *TPS-e/f* subfamily of *P. equestris*, *P. bellina* and other plants. *PbTPS3* and *PbTPS4* from *P. bellina*, *LIS* from *C. concinna*, *LIS2* from *C. concinna*, *S-LIS* from *C. breweri*, *AFS1* from *A. deliciosa*, and *GES* from *A. thaliana*. The red boxes indicate the three conserved motifs of DDXXD and NSE/DTE, respectively. The red line is the conifer diterpene internal sequence (CDIS). *PbTPS3* and *PbTPS4* share 62.4% identity.

Supplementary Figure S9. (A) Expression of *PbTPS3* vegetative organs with high expression in leaf but very low expression in root and stalk. (B) VOCs emitted from leaf were mostly (β)-cis-oicmene. (C) Structure of (β)-cis-oicmene.

Supplementary Figure S10. The binding domains of plant defense transcription factor on the promoter sequences of *PbTPS3* and *PbTPS4*. (A) The gene promoter was indicated as 2,000 bp upstream from the translation start site. (B) WBOXATNPR1 is marked with a blue box, and WRKY71OS is marked with a green box.

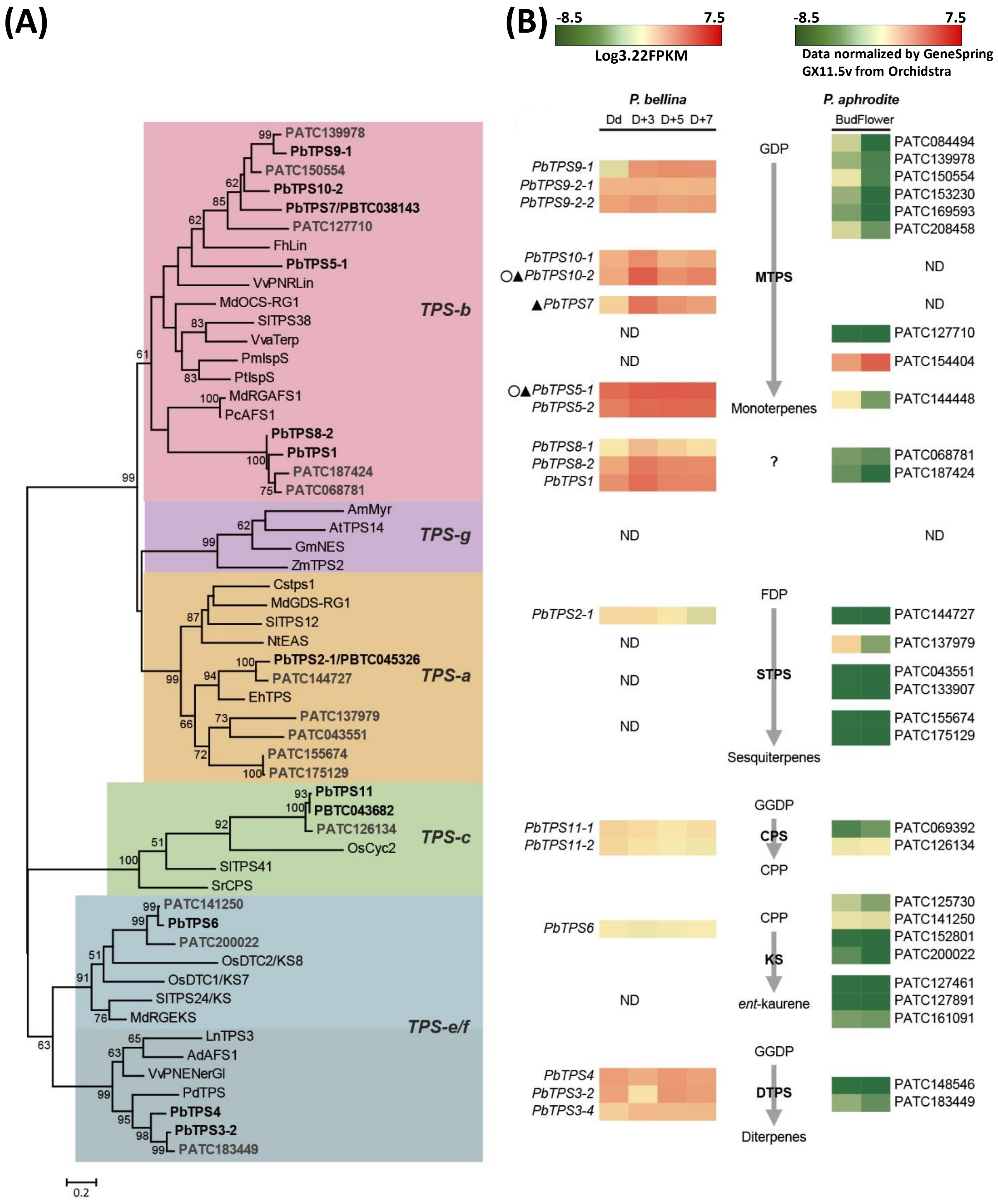

Huang *et al.*, Supplementary Figure S1

(A)

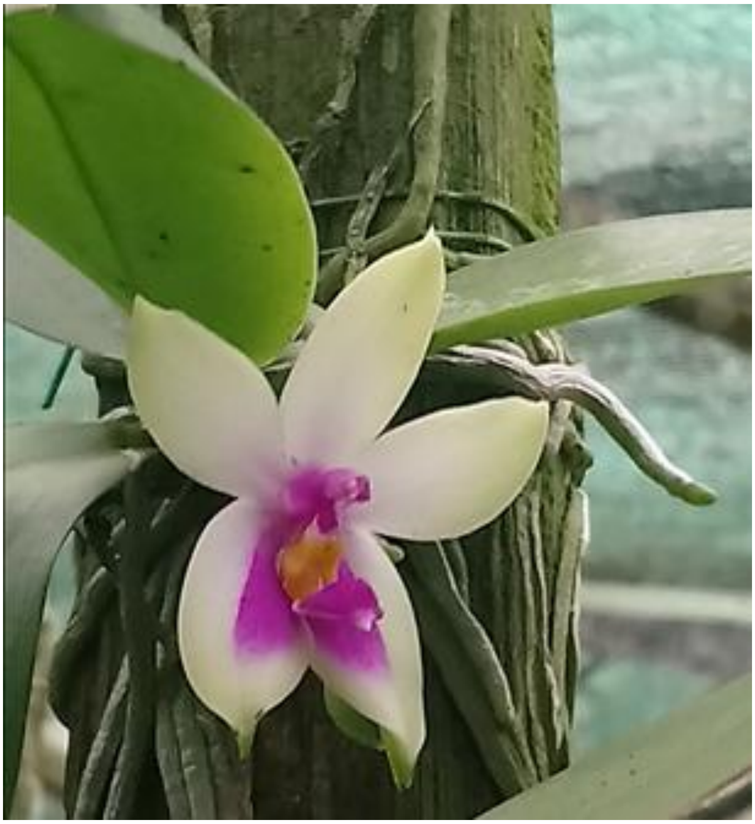

(B)

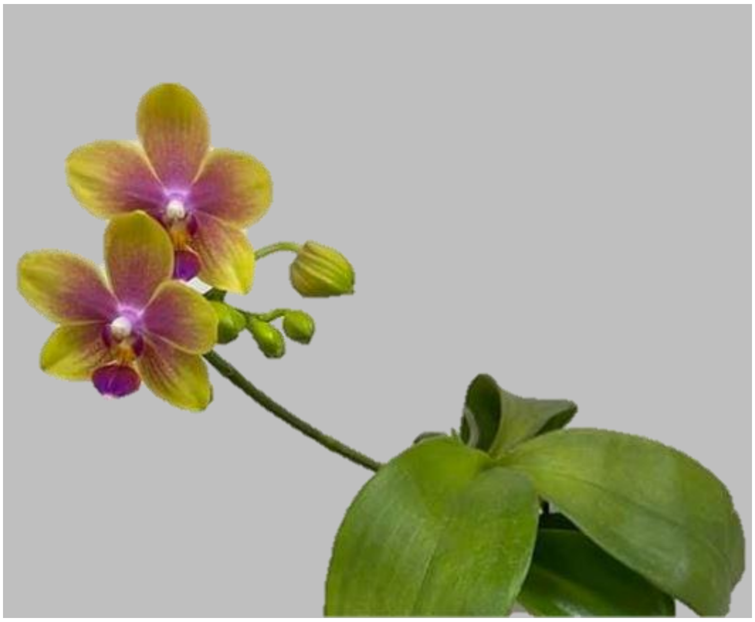

Huang *et al*., Supplementary Figure S2

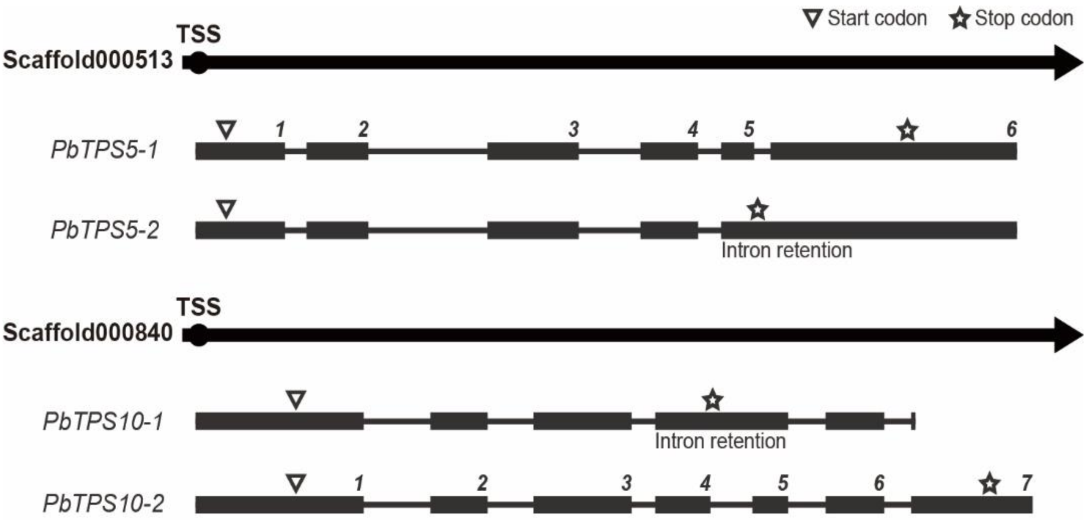

Huang *et al*., Supplementary Figure S3

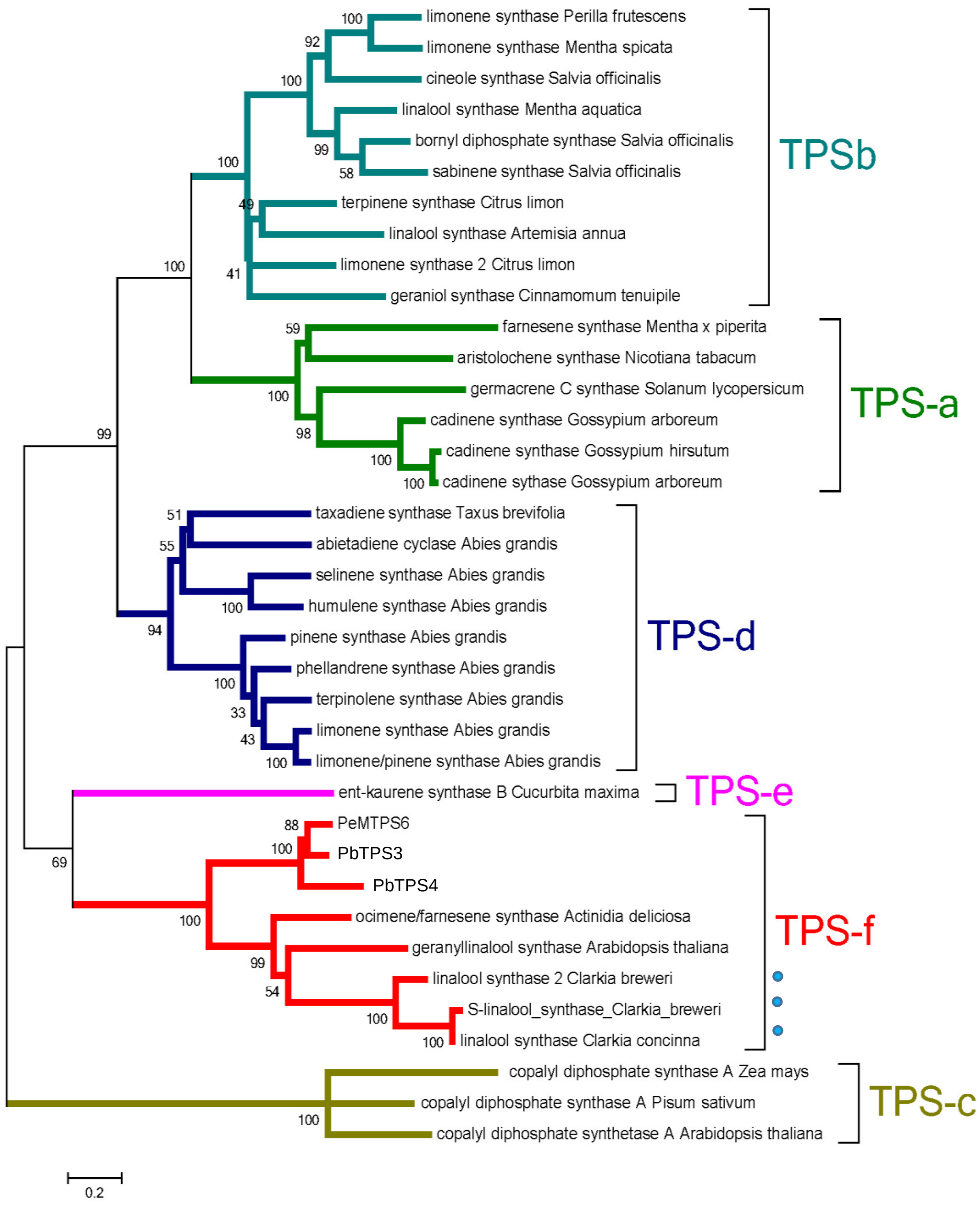

Huang *et al.*, Supplementary Figure S4

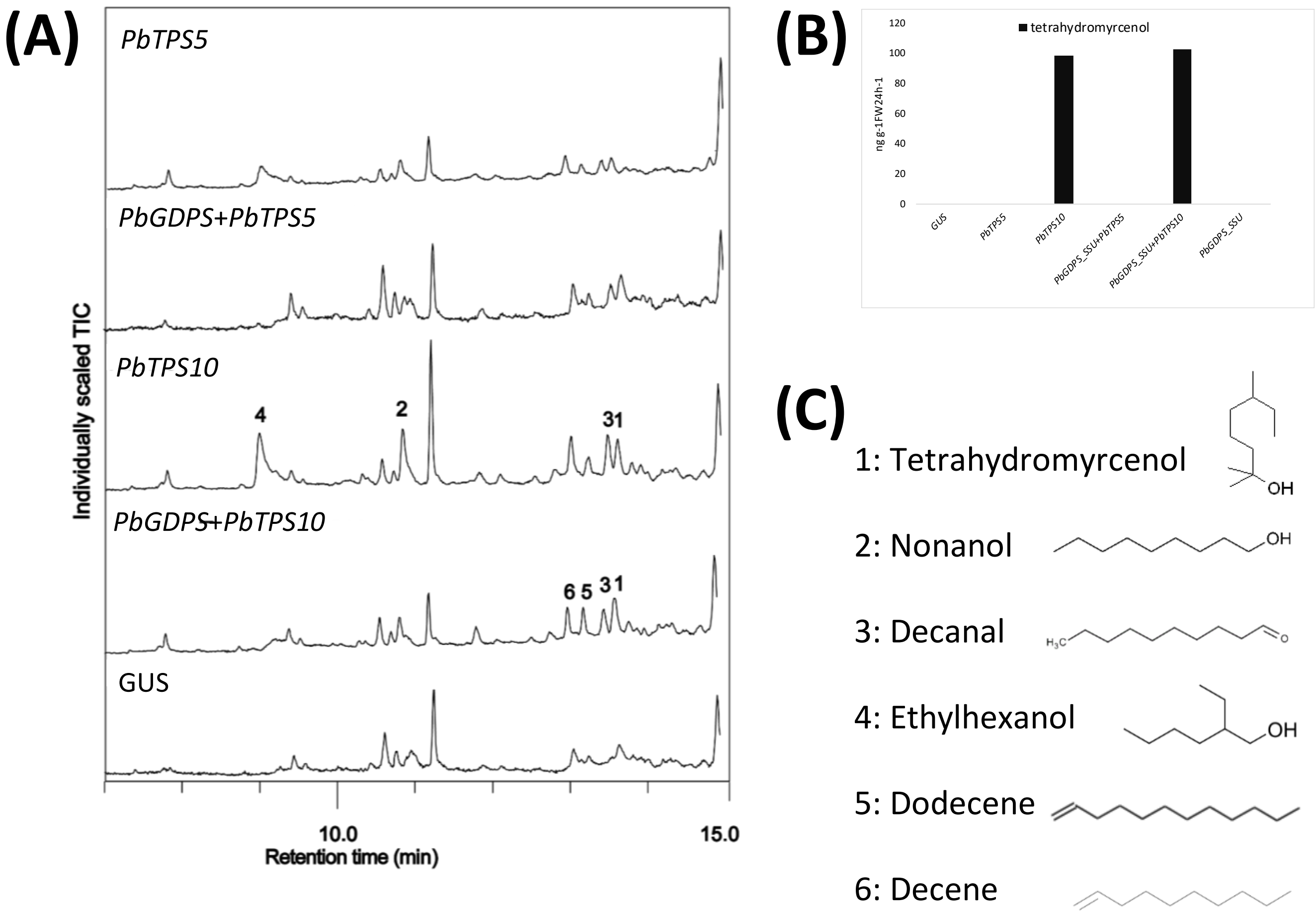

Huang *et al*., Supplementary Figure S5

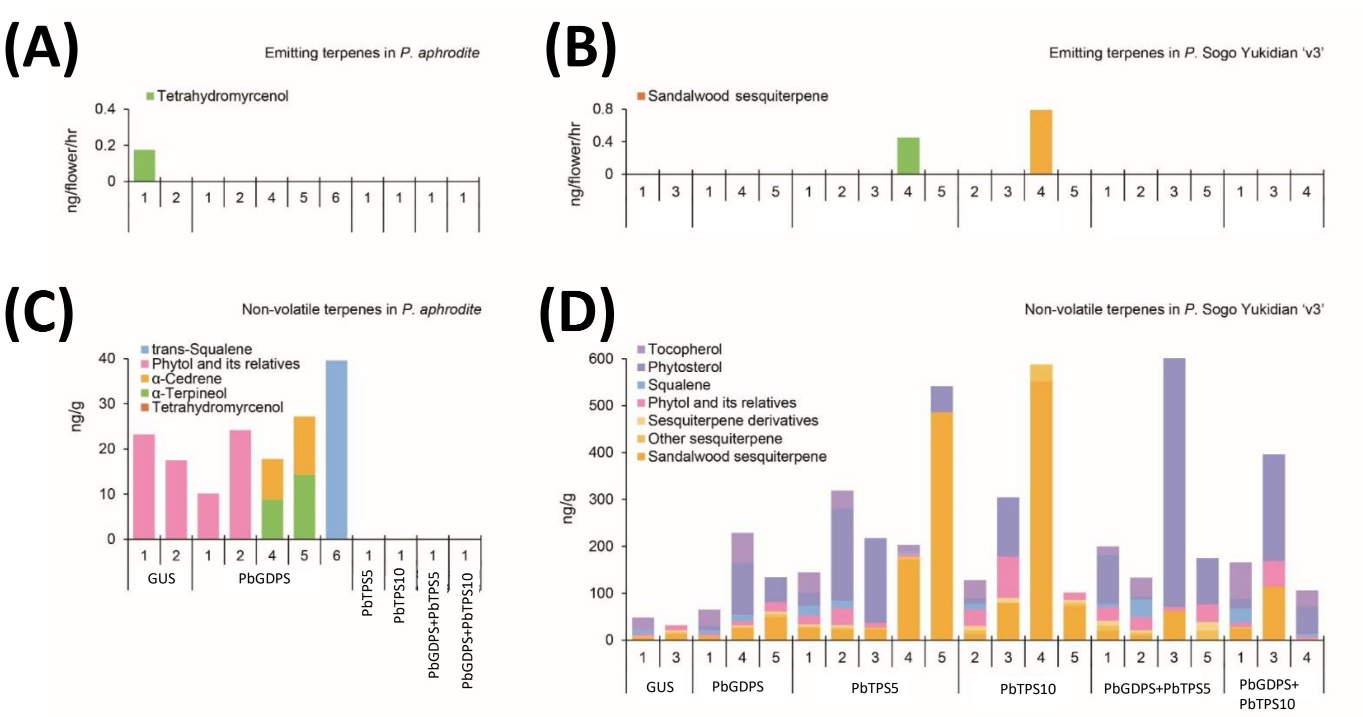

Huang *et al*., Supplementary Figure S6

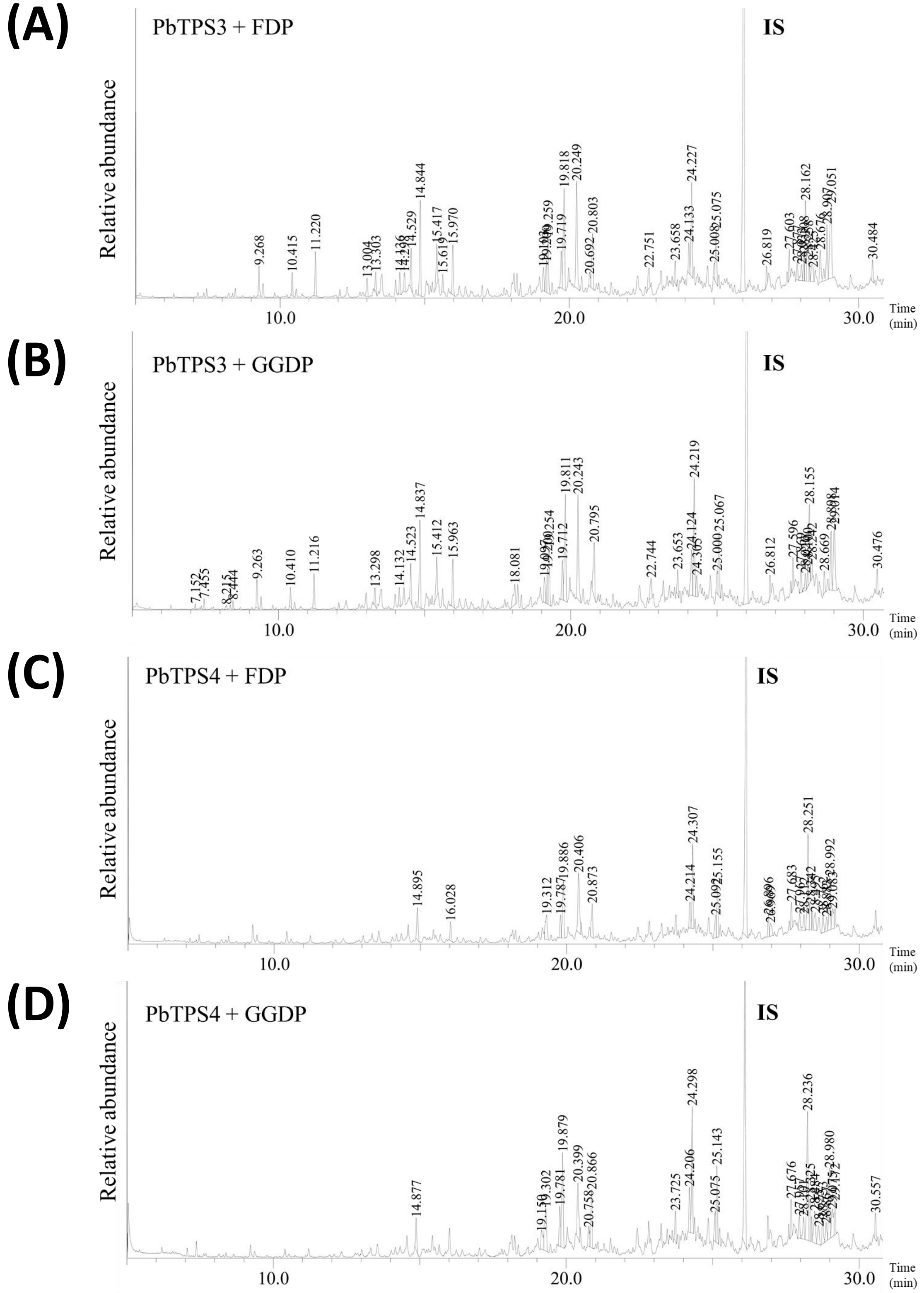

Huang *et al*., Supplementary Figure S7

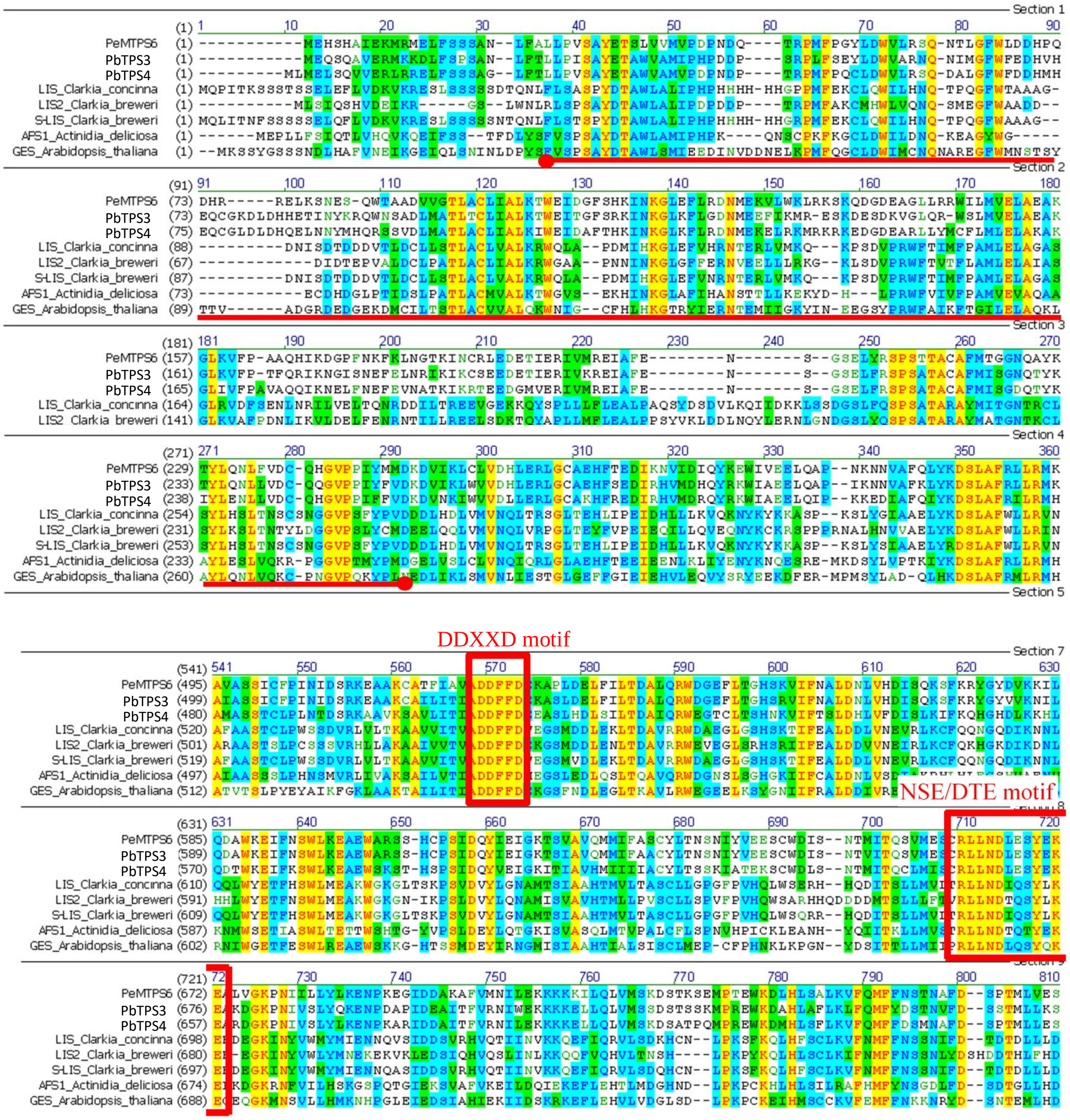

Huang *et al*., Supplementary Figure S8

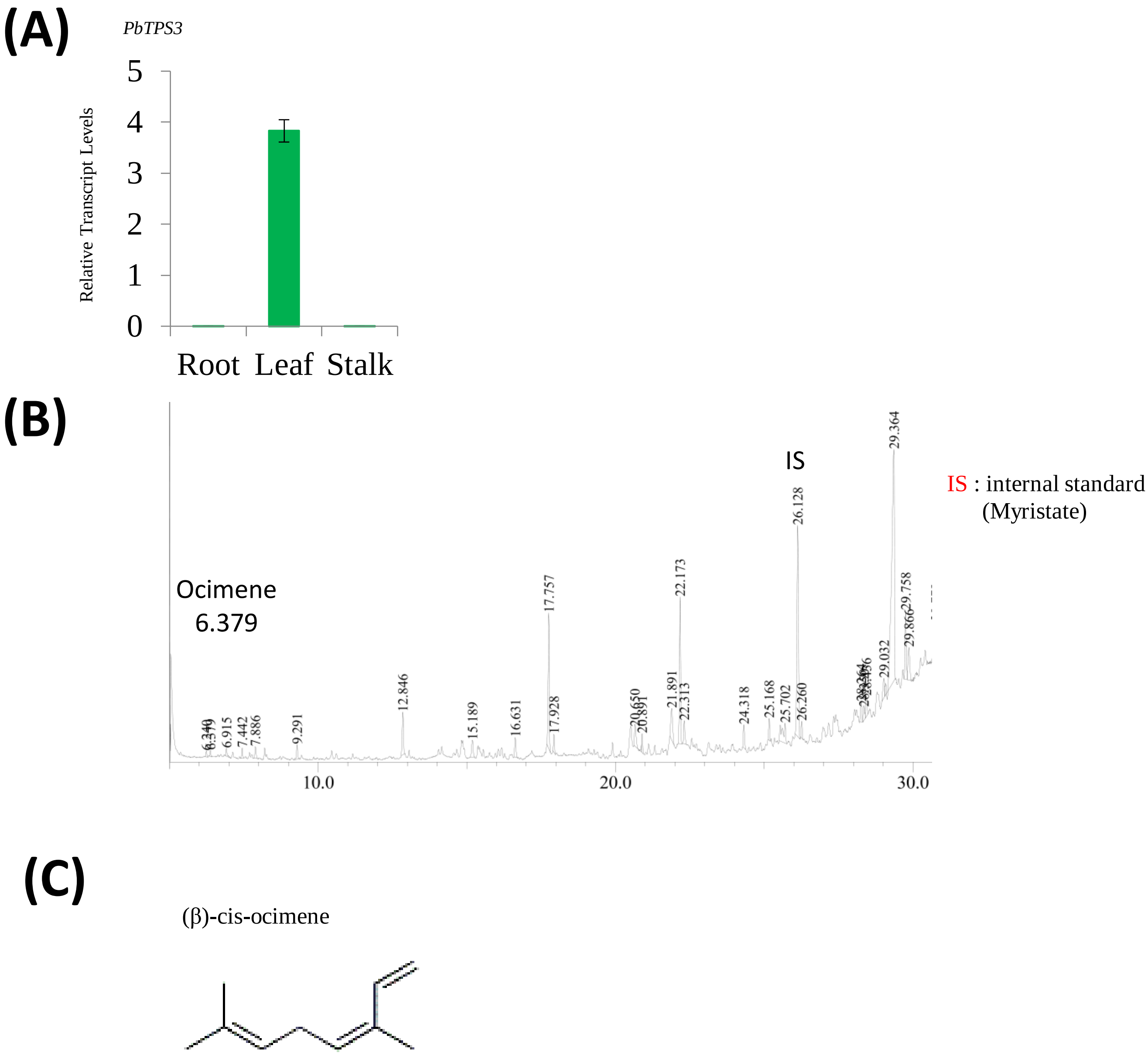

Huang *et al*., Supplementary Figure S9

(A)

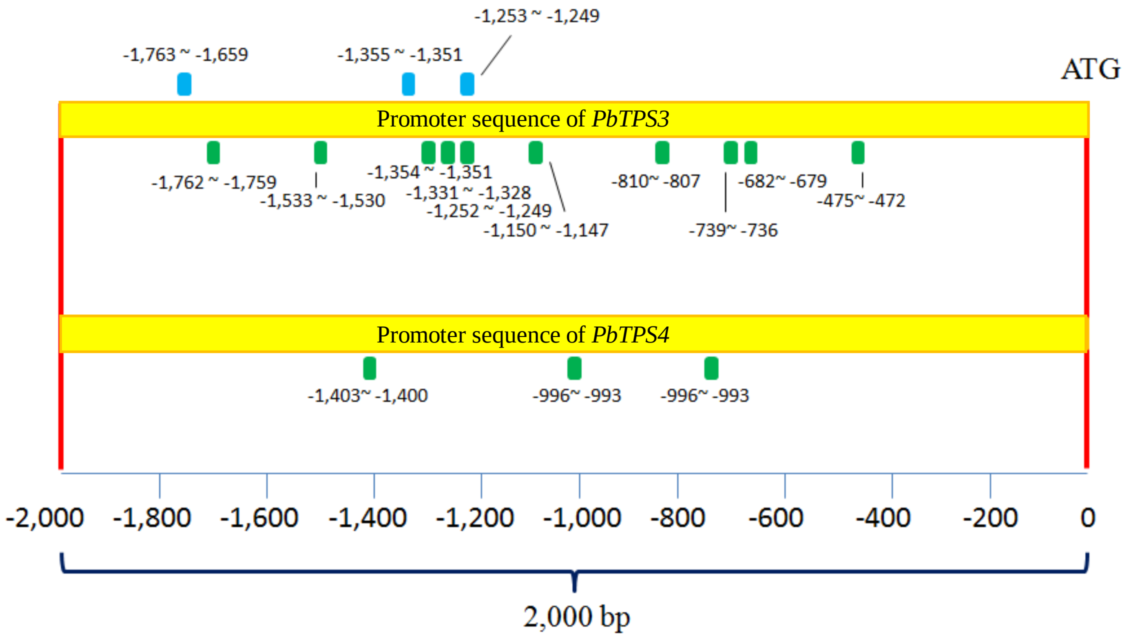

(B)

	Domain name	Sequence	Function
■ (blue)	WBOXATNPR1	TTGAC	Specifically by salicylic acid (SA)-induced WRKY DNA binding
■ (green)	WRKY71OS	TGAC	Early nuclear events in plant defence signalling

Huang *et al.*, Supplementary Figure S10
